# Citrullination of VDAC3 by PAD2 restricts mitochondrial metabolism and suppresses macrophage reprogramming in sepsis

**DOI:** 10.7150/ijbs.126368

**Published:** 2026-09-11

**Authors:** Liujiazi Shao, Chao Quan, Tao Dong, Yuchen Chen, Xin Yu, Wenlu Ouyang, Dan Qi, Jeffrey Chu, Erxi Wu, Zequan Yang, Jianjie Ma, Yongqing Li

**Affiliations:** 1 Department of Surgery, University of Michigan Health System, Ann Arbor, United States.; 2 Department of Anesthesiology, Beijing Friendship Hospital, Capital Medical University, Beijing, China.; 3 Department of Surgery, Division of Surgical Science, University of Virginia, Charlottesville, United States.; 4 Neuroscience Institute and Department of Neurosurgery, Baylor Scott & White Health, Temple, Texas, United States.; 5 Metware Biotechnology Inc., Woburn, MA, United States.; 6 Department of Neurosurgery, Baylor College of Medicine, Temple, Texas, United States.; 7 Texas A&M University Colleges of Medicine and Pharmacy, College Station, United States.; 8 Cancer Research Center and Department of Internal Medicine, Dell Medical School, the University of Texas at Austin, Austin, Texas, United States.

**Keywords:** *Pad2* knockout, VDAC3, α-ketoglutarate, M2 macrophage polarization, glutamine

## Abstract

Sepsis is a life-threatening inflammatory disorder characterized by immune dysregulation and organ failure, and while peptidylarginine deiminase-2 (PAD2) regulates immune responses via citrullination, its role in immunometabolism remains unclear. Here we identify a PAD2-dependent mechanism that impairs mitochondrial glutamine metabolism and macrophage polarization during sepsis. Through single-cell RNA sequencing of bronchoalveolar lavage fluid from septic mice, we found that *Pad2*^-/-^ promotes M2 macrophage polarization, reduces lung injury, and improves survival. Using mass spectrometry-based citrullinomics, we identified voltage-dependent anion channel-3 (VDAC3) as a PAD2 substrate, and further demonstrated that PAD2-mediated citrullination at residue R252 suppresses glutamine transport and α-ketoglutarate (α-KG) production, thereby impairing oxidative phosphorylation (OXPHOS) and M2 reprogramming. Notably, substitution of R252 with alanine recapitulated *Pad2*^-/-^phenotypes, and consistent with this, *Pad2*^-/-^ mice exhibited enhanced OXPHOS, attenuated inflammation, and improved outcomes in a Pseudomonas aeruginosa-induced model of sepsis and lung injury. Collectively, these findings establish the PAD2-VDAC3-glutamine axis as a key immunometabolic checkpoint that links post-translational modification to mitochondrial metabolism and macrophage function, suggesting that targeting this pathway may offer a promising strategy to restore immune balance in sepsis.

## Introduction

Sepsis is a life-threatening syndrome that arises from a dysregulated host response to infection and remains a leading cause of mortality worldwide 1. During sepsis, the immune system undergoes a dynamic transition from hyperinflammation to immune paralysis, where the failure to resolve inflammation drives tissue damage, organ dysfunction, and poor clinical outcomes 2. Macrophages play a central role in orchestrating both the propagation and resolution of inflammation, placing them at the core of immune homeostasis in sepsis.

Macrophage polarization into pro-inflammatory (M1) or anti-inflammatory (M2) phenotypes is tightly regulated by a complex interplay of environmental and metabolic signals 3. M1 macrophages depend on glycolysis and promote pathogen clearance and inflammation, whereas M2 macrophages rely on oxidative metabolism to perform anti-inflammatory functions, tissue repair, and the restoration of homeostasis 4,5. Recent studies have shown that promoting M2 polarization accelerates the resolution of inflammation and improves survival in preclinical models of sepsis, emphasizing the importance of immunometabolic in innate immunity 6. Mitochondria act as key hubs for immunometabolic signaling in M2 macrophages. Through oxidative phosphorylation (OXPHOS), they generate adenosine triphosphate (ATP), maintain redox balance, and produce key metabolites such as α-ketoglutarate (α-KG), which promotes M2 polarization via epigenetic and metabolic mechanisms 7. Impairing mitochondrial metabolism disrupts M2 differentiation, skewing macrophage function toward a sustained pro-inflammatory state.

Peptidylarginine deiminase 2 (PAD2) is a calcium-dependent enzyme that catalyzes the conversion of arginine to citrulline, modulating protein structure and function via post-translational citrullination 8. Although PAD2 is highly expressed in monocytes and macrophages, its role in innate immune metabolism remains poorly defined. We recently demonstrated that genetic deletion of *Pad4*, another PAD isoform, does not to improve sepsis outcomes. In contrast, *Pad2^-/-^* mice or selective pharmacologic inhibition of PAD2 significantly enhance survival and reduce systemic inflammation-highlighting a distinct, non-redundant role for PAD2 in the pathogenesis of sepsis 9-12. During infection, macrophages increasingly depend on glutamine, a key metabolic substrate that fuels mitochondrial respiration and α-KG production. Glutamine-derived α-KG is a key metabolite that promotes M2 polarization through both metabolic and epigenetic mechanisms 7,13. However, whether PAD2 interferes with mitochondrial glutamine utilization and thereby alters macrophage polarization during sepsis remains unknown.

In the present study, we identify voltage-dependent anion channel 3 (VDAC3), a mitochondrial outer membrane protein that regulates metabolite exchange, as a previously unrecognized substrate of PAD2. We show that PAD2 catalyzes site-specific citrullination of VDAC3 at arginine 252, thereby impairing mitochondrial glutamine transport. Loss of PAD2 or substitution of R252 enhances glutamine uptake, increases α-KG accumulation, elevates OXPHOS activity, and promotes M2 macrophage polarization. This immunometabolic reprogramming attenuates inflammation and improves survival in septic mice. Our findings uncover a PAD2-VDAC3 citrullination axis as a novel regulatory checkpoint that links post-translational modification to mitochondrial metabolism and macrophage function. Targeting this pathway may offer a therapeutic strategy to restore immune balance and promote recovery in sepsis.

## Materials and Methods

### Study design

This study investigated the role of PAD2 in regulating macrophage metabolism and polarization during sepsis. Using both *in vivo* and *in vitro* approaches, we examined the PAD2-VDAC3 axis in immunometabolic reprogramming. A *Pseudomonas aeruginosa* (PA)-induced sepsis model was used to assess survival, lung injury, and immune responses in WT and *Pad2*^-/-^ mice. Single-cell RNA sequencing (ScRNA-Seq) of bronchoalveolar lavage fluid (BALF) cells was performed to analyze macrophage polarization. PAD2 targets were identified via mass spectrometry-based citrullinomics. To assess functional effects, we introduced a VDAC3 R252A mutation into RAW 264.7 macrophages and evaluated mitochondrial glutamine uptake, α-KG production, and polarization status. OXPHOS was analyzed via Seahorse assays in *Pad2*^-/-^ BMDMs. Inflammatory markers, survival, and metabolic parameters were measured to determine the impact of *Pad2*^-/-^ deficiency. All experiments included appropriate controls and replicates, with validation through complementary methods.

### Mice and sepsis model

WT C57BL/6 and *Pad2*^-/-^ male mice (8-12 weeks old) were used in this study. WT mice were purchased from the Jackson Laboratory (Bar Harbor, ME, USA) and acclimatized in our pathogen-free animal facility for 3 days prior to any experimental procedures. *Pad2*^-/-^ mice were kindly provided by Dr Scott Coonrod (Cornell University, Ithaca, New York, USA). The mice were housed in a specific pathogen-free environment with a controlled temperature of 23±1.5°C and relative humidity of 70±20%. Experimental protocols and animal care methods were reviewed and approved by the Institutional Animal Care and Use Committee (IACUC) at the University of Michigan and performed according to their guidelines (PRO00011567).

Mice were intranasally administered PA (19660; ATCC) solution to induce PA-induced sepsis. Briefly, a PA solution was prepared at a concentration of 8.25 × 10^7^ CFU/mL in PBS. The mice were anesthetized with ketamine and xylazine and then held in a vertical position. Subsequently, 15 μL of the PA solution was instilled into each nostril (30 μL total) to achieve a final bacterial load of 2.5 × 10^6^ CFU per mouse. Mice inoculated with sterile PBS served as sham controls. For the nonsurvival studies, the mice were euthanized with CO_2_ 24 hours after inoculation. In survival studies, WT and *Pad2*^-/-^ mice were monitored for 10 days, after which they were euthanized with CO_2_ either at the designated endpoint of observation or when they were found moribund.

### Histological assessment of lung injury

The lungs of the mice were harvested either 24 hours after sham treatment or following PA infection. The tissues were fixed in 4% neutral-buffered formaldehyde and subsequently embedded in paraffin. Lung tissue sections were stained with H&E and graded by a board-certified pathologist who was blinded to the experimental conditions.

### Sample preparation and scRNA-seq

BALF samples were collected from both WT and *Pad2*^-/-^ mice after PA infection and from the sham control. Samples from 3 mice per group were pooled (n = 3 mice/sample) for BALF cell isolation. Samples were centrifuged for 5 minutes at 400g and 4 °C. The cell pellet was then resuspended in 500 μL of RBC lysis buffer and incubated for 5 minutes at room temperature. Next, 500 μL of cold PBS was added to dilute the RBC lysis buffer, and the mixture was centrifuged for 5 minutes at 400 g and 4 °C. The resulting single cells were resuspended in PBS containing 1% weight/volume FBS, and cell viability was determined using automated cell counters (Invitrogen). The single-cell suspension was thoroughly mixed and loaded onto a 10X Chromium system to capture no more than 10,000 single cells using the Chromium Next GEM Single Cell 3′ GEM, Library & Gel Bead Kit (10X Genomics). The cells were partitioned into Gel Beads in a Chromium instrument. DNA amplification and library construction were performed via cell lysis and barcoded reverse transcription of RNA. The resulting libraries were sequenced via an Illumina HiSeq 4000 next-generation sequencing platform. Data quality analysis and mapping to ensemble gene symbols were conducted via Cell Ranger (10X Genomics).

### ScRNA-seq data processing

The Cell Ranger output data were imported into the Seurat R package (version 4.3.0) for unsupervised clustering analysis. Prior to clustering, filtering procedures were implemented to eliminate multiples and damaged cells, while sources of variation deemed uninformative were regressed out. Identification of variable genes was achieved through iterative selection based on the dispersion versus average expression profile of each gene. Normalization of gene expression values within individual cells was conducted via the Log Normalize method, with a scale factor of 10,000. A total of 2,000 highly variable features were identified for dimensionality reduction. Batch effects were corrected using the Harmony algorithm. Dimensionality reduction and visualization of the data were performed via principal component analysis (PCA) and UMAP, incorporating the top 20 principal components. The parameters for UMAP were set to dims = 1:20. The cells were subsequently clustered via an unsupervised clustering approach with default parameters for the Seurat package (resolution = 0.5). Cluster-specific marker genes were identified utilizing the FindAllMarkers function in Seurat, with criteria set at a p-value < 0.01 and log (fold change) > 0.25 within the target cluster. Visualization of gene expression patterns across cell clusters was accomplished via UMAP plots and dot plots generated with functions available in the Seurat package. Gene expression values were normalized using Gapdh as a reference by dividing each gene's expression by its corresponding Gapdh expression. The expression values were z-score normalized across all genes. Visualization was performed via dot plots, where average expression was color-scaled, and percentage expression was size-scaled, comparing the WT Sepsis and *Pad2^-/-^* Sepsis samples. For gene module scoring, the AddModuleScore function was used for a custom gene list. The resulting scores for the WT and *Pad2^-/-^* sepsis samples were visualized via boxplots, with statistical significance determined via the Wilcoxon test.

### RNA preparation and real-time PCR

RNA isolation was performed according to standard procedures (Qiagen kit). Total RNA was extracted from cells via a RNeasy Mini kit. Quantitative real-time PCR was conducted with SYBR Green (Qiagen) and Quant Studio 3 system. The gene expression levels measured in this study were normalized to the expression level of the housekeeping gene *Gapdh* and calculated via the 2^-ΔΔCT^ method. The specific primer sets for each target gene are listed in Table.

### Bone marrow-derived macrophages (BMDMs) isolation and differentiation

BMDMs were isolated for *in vitro* experimentation. Tibiae and femurs were obtained from both WT and *Pad2*^-/-^ mice. Bone marrow cells were collected and seeded in 75 mm^2^ petri dishes containing IMDM supplemented with 20% FBS, 1% penicillin/streptomycin, and 30% L929 cell supernatant. The L929 cell supernatant was generated by incubating L929 cell fibroblasts in IMDM with 10% FBS for 6 days to produce macrophage CSF (M-CSF). After 7 days, the BMDMs were harvested and diluted in Opti-MEM (Thermo Fisher Scientific) to the desired concentrations. For experiments involving LPS treatment, BMDMs were exposed to 200 ng/mL PA-deprived LPS in Opti-MEM for 24 hours, while control BMDMs were treated with Opti-MEM alone for the same duration.

### Untargeted metabolomics

The cell pellets were removed from -80 °C and maintained on wet ice during processing. 200 μL of 80% methanol in water containing internal standards was added to each microtube for quality control purposes. The samples were sonicated using a Branson 450 Probe Sonifier at 20% power, and a 20% duty cycle for 10 seconds, then placed on wet ice. The microtubes were vortexed and incubated at 4°C for 5 minutes for complete metabolite extraction. Following centrifugation at 16,000 g for 5 min at 4 °C, 200 µL of the resulting supernatant was transferred to autosampler vials. A pooled sample was generated by combining 25 µL of supernatant from each individual sample. All the samples were dried under a gentle nitrogen stream at room temperature until drying. For LC‒MS analysis, the samples were reconstituted in 20% methanol in water.

For the Reversed-Phase Liquid Chromatography-Mass Spectrometry (RPLC-MS) analysis, samples were analyzed using an Agilent 1290 Infinity II/6545 Q-TOF MS system with a Jet Stream Ionization (ESI) source (Agilent Technologies, Inc., Santa Clara, CA, USA), equipped with a Waters Acquity HSS T3 column (1.8 µm, 2.1x 50 mM; Waters Corporation, Milford, MA, USA). Each sample was analyzed twice, once in positive ion mode and once in negative ion mode. Mobile phase A was 100% water with 0.1% formic acid and mobile phase B was 100% methanol with 0.1% formic acid. The gradient for both positive and negative ion modes was as follows: 2% B (0 min), 75% B (20 min), 98% B (22 min), 98% B (30 min), and 2% B (30.1 min) was used. The column was then reconstituted for 7 min with 2% B before being moved to the next injection. The flow rate was 0.46 mL/min, and the column temperature was 40 °C. The injection volumes for the positive and negative modes were 5 µL and 8 µL, respectively. The source parameters were a drying gas temperature of 350 °C, drying gas flow rate of 10 L/min, nebulizer pressure of 30 psi, sheath gas temperature of 350 °C and flow rate of 11 L/min, and capillary voltage of 3500 V, with internal reference mass correction. Data analysis for this platform follows a hybrid targeted/non-targeted approach. Semi-quantitative data for known compounds were obtained by manual integration using Profinder v8.00 (Agilent Technologies, Santa Clara, CA, USA). Metabolites were identified by matching the retention time (+/- 0.1 min), mass (+/- 10 ppm) and isotope profile (peak height and spacing) to those of authentic standards. Non-targeted data analysis was performed via Agilent's MassHunter Find by Molecular Feature workflow (v7.0) with recursion via Agilent's Mass Profiler Pro (v8.0).

### Targeted metabolomics (amino acid analysis and TCA analysis)

The cell pellets were retrieved from -80 °C and maintained on wet ice during processing. To each microtube, 400 µL of 1:1:1:1 methanol:acetone:acetonitrile:water containing a mixture of seventeen ^13^C-labeled amino acids (Sigma 96738), supplemented with ^13^C_5_ glutamine, ^15^N_2_-asparagine, and ^15^N_2_-tryptophan, all at 500 nM (final), as well as eight TCA cycle internal standards at 250 nM (Cambridge Isotopes MSK-TCA1-IS), was added. The samples were then probe-sonicated at 20% power with a 20% duty cycle for 10 seconds and placed on wet ice. The microtubes were vortexed and allowed to incubate at 4 °C for 5 minutes to complete metabolite extraction. The samples were subsequently centrifuged at 16,000 RPM for 5 min at 4 °C. A pooled sample was created by combining 12.5 µL of each sample into an autosampler vial for amino acid (AA) analysis, and this step was repeated for TCA analysis. All the samples were dried under a gentle nitrogen stream at room temperature and reconstituted in 37.5 µL of 80/20 water/methanol for LC-MS analysis.

A standard stock of all 20 proteinogenic amino acids was prepared in LC-MS grade water at a concentration of 25 µM for each AA. Calibration standard solutions were then prepared by diluting this stock to concentrations of 0, 0.25, 0.833, 2.5, 8.3 and 25 µM, for a total volume of 100 µL each. The standard solutions were each further diluted by the addition of 400 µL extraction solvent (with internal standards), then transferred to autosampler vials and dried, and reconstituted in 100 µL 20% methanol in water. A standard stock of TCA metabolites (MSK-TCA (unlabeled) mix at 200 µM) was prepared in LC-MS grade water to a final concentration of 10 µM. Calibration standard solutions were then prepared by diluting this stock to concentrations of 0, 0.1, 0.3, 1, 3 and 10 µM, for a total volume of 100 µL each. The standard solutions were each further diluted by the addition of 400 µL extraction solvent (with internal standards), then transferred to autosampler vials and dried, and reconstituted in 100 µL 20% methanol in water.

Amino acid LC-MS/MS analysis was performed via liquid chromatography-tandem mass spectrometry on an Agilent 6410 triple quadrupole system equipped with an Agilent 1200 Binary pump. Chromatographic separation was performed by hydrophilic interaction liquid chromatography (HILIC) on an Intrada Amino Acid column, 100 mm in length x 3 mm in diameter. Mobile phase A was 100 mM ammonium formate in 80/20 water/acetonitrile and mobile phase B consisted of 0.3% formic acid in acetonitrile. The gradient was as follows: hold 20% B for 0-4min; linear ramp 20-100% B from 4min-14 min, hold 100% B for 14-16 min; linear ramp 100-20% B for 16-16.1 min, hold 20% B for 16-20 min. The flow rate was 0.6mL/min, the column temperature was 37°C, and the injection volume was 5 µL. MS parameters were as follows: positive ion mode, gas temperature 325 °C, gas flow rate 10L/min, nebulizer pressure 40 psi, and capillary voltage 4000 V. Detection was performed in multiple reaction monitoring mode with parameters as indicated in the attached table.

TCA LC-MS analysis was performed on an Agilent system consisting of an Infinity Lab II UPLC coupled with a 6530 Q-TOF mass spectrometer (Agilent Technologies, Santa Clara, CA) using a Jet Stream ESI source in negative mode. The following source parameters were used: gas temperature 250 ºC, gas flow 13 L/min, nebulizer 35 psi, sheath gas temperature 325 ºC, sheath gas flow 12 L/min, capillary 3500 V, and nozzle voltage 1500 V.

### Identification of citrullination sites by LC-MS/MS

BALF samples from three mice per group were pooled to generate one biological replicate samples (n = 3 mice/sample). The samples were centrifuged at 400 g at 4°C for 5 minutes to pellet cells, which were then treated with RBC lysis buffer (eBioscience) to remove red blood cells. The isolated BALF cells were suspended in RIPA buffer containing a protease inhibitor cocktail and heated at 95 °C for 15 minutes. Protein concentration was determined via Qubit fluorometry (Invitrogen). Subsequently, 10 µg of protein from each sample was loaded onto a 10% Bis-Tris NuPage Mini-gel (Invitrogen) using the MES buffer system. Gel electrophoresis was performed until the dye front reached ~2 cm, and the gel lanes were then cut into 10 equally sized bands for in-gel digestion.

In-gel digestion was carried out with trypsin via a robot (DigestPro, CEM), involving washing with 25 mM ammonium bicarbonate followed by acetonitrile, reduction with 10 mM dithiothreitol at 60 °C, alkylation with 50 mM iodoacetamide at room temperature, and digestion with sequencing grade trypsin (Promega) at 37 °C for 4 hours. The digestion process was quenched with formic acid, and the supernatant was directly analyzed. Half of each digested sample was subjected to nano LC-MS/MS via a Waters M-Class LC system coupled to a ThermoFisher Exploris 480 mass spectrometer. Peptides were loaded onto a trapping column and eluted over a 75 μm analytical column packed with XSelect CSH C18 resin (Waters). The column temperature was maintained at 55 °C via a column heater (Sonation), and the mass spectrometer was operated in data-dependent mode with the Orbitrap set at 60,000 FWHM and 15,000 FWHM for MS and MS/MS, respectively, with a 3-second cycle for both MS and MS/MS. Each sample was analyzed for 5 hours. Mascot with trypsin/P enzyme parameters was used for the data search against the SwissProt Mouse database appended with *Pseudomonas aeruginosa*. A differential modification of 0.984 on arginine was specified to account for citrullination by PAD2. Data were filtered and analyzed via Scaffold 5 proteome software (version: 5.3.3).

### Oxygen consumption rate (OCR)/ Extracellular acidification rate (ECAR) analysis

OCR and ECAR were determined with an XFe96 extracellular flux analyzer (Agilent Technologies) as described in the manufacturer's protocol. A total of 10000 cells were seeded per well in 96-well micro cell culture plates (Agilent Technologies) in DMEM with 10% FBS and incubated at 37°C overnight in a 5% CO_2_ incubator. The next day, the growth medium was replaced with phenol red- and bicarbonate-free DMEM (pH 7.4). Cells were incubated at 37 °C in a non-CO_2_ incubator to equilibrate the CO_2_ level in the atmosphere.

OCR and ECAR were measured under baseline conditions and in response to sequential administrations of metabolic modulators, depending on the assay type. For Cell Mito Stress Test Kit, the final concentrations of the compounds used were as follows: oligomycin (2 μM), FCCP (carbonyl cyanide-p-trifluorome-thoxyphenylhydrazone, 2 μM) and rotenone/antimycin A (0.5 μM/0.5 μM). For the Glycolysis Stress Test Kit, glucose (10 mM), oligomycin (2 μM), and 2-DG (2-deoxyglucose, 50 mM). Each measurement cycle consisted of 3 min of mixing, 0 min of waiting, and 3 min of measurement. Following the Seahorse analysis, nuclei were stained with Hoechst dye, and the XFe96 microplate was transferred to a BioTek Instrument′s Cytation 5 system. Hoechst-stained fluorescent nuclear images were captured with autofocus via Gen5 software. The number of nuclei was determined via the Cell Analysis function in Gen5 software and used to normalize the Seahorse assay data in WAVE software (Agilent Technologies).

### Protein extraction and western blotting

The cells were washed twice with PBS and sonicated on ice in RIPA buffer supplemented with protease inhibitor cocktail for 30 minutes. The protein concentration was measured by BCA assay. The samples were mixed with 4×Laemmli buffer, subjected to SDS-PAGE and transferred to nitrocellulose membrane via the Bio-Rad Western Blotting System. After being blocked in TBST buffer containing 5% milk, the membrane was incubated with primary antibody in 3% bovine serum albumin overnight at 4°C. The next day, the membranes were subsequently incubated with either anti-mouse or anti-rabbit secondary antibodies for 1 hour at room temperature, visualized via a Bio-Rad ChemiDoc™ Imaging System. The band intensity was quantified by ImageJ software and normalized to β-actin or Tubulin.

### Purification of mitochondria from cells and measurement of mitochondrial glutamine uptake

The mitochondria were purified following the manufacturer's instructions (Thermo Scientific, 89874). After purification, the mitochondria were resuspended in KPBS buffer (136 mM KCl, 10 mM KH_2_PO_4_, pH 7.2 in deproteinized and sterilized water); the pH was adjusted with KOH to prevent sodium sensitivity. For the mitochondrial glutamine uptake assay, purified mitochondria were further diluted with KPBS solution. Next, 50 µL of the mitochondrial suspension was dispensed into each well of a poly-L-lysine-coated 96-well plate. The microplate was then placed in a centrifuge equipped with a swinging bucket microplate adaptor and spun at 2,000 g for 20 minutes. After centrifugation, the supernatant was discarded, and 100 µL of prewarmed KPBS solution was added to each well. The initial 100 µL KPBS in each well was aspirated, and 100 µL KPBS containing radiolabeled glutamine ([L-[^3^H] Gln) (Perkin Elmer) (3 µCi/ml) was added. All measurements were conducted at 37°C for the designated time. Following incubation, the liquid in the wells was aspirated, and 200 µl DPBS was gently added along the well wall. After gentle shaking, the liquid in the wells was aspirated. Subsequently, 200 µL of lysis solution (RIPA lysis buffer plus 0.1% SDS) was added to each well for mitochondrial lysis on ice for 30 minutes. The lysate was transferred to 20 ml glass vials, mixed with 10ml of Cocktail buffer (ECONO), and counted via TRI-CARB 4910TR Liquid Scintillation Counter (PerkinElmer). The mitochondrial protein concentration was determined using a BCA assay kit. Radiolabeled glutamine uptake was calculated based on counts per minute (CPM) per sample and normalized to the mitochondrial protein content.

### Stochastic Optical Reconstruction Microscopy (STORM) super-resolution imaging

STORM super-resolution imaging was performed at the Single Molecule Analysis in Real-Time (SMART) Center of the University of Michigan on a bespoke TIRF microscope built off an Olympus IX81 inverted microscope base. BMDMs were cultured on #1.5-thickness coverglass-bottomed cell culture dishes. After preparing the cells, they were fixed in 2% paraformaldehyde (PFA) for 1 hour at 4 °C, followed by a rinse in 100 mM glycine solution for 1 hour at 4 °C. The samples were then washed with phosphate-buffered saline (PBS) for 30 minutes and stored in PBS until further processing. The slides were incubated in an optimized blocking solution containing 5% normal donkey serum and 0.3% Triton X-100 in PBS for 1 hour. Following this, the slides were incubated with primary antibody, diluted in the blocking solution, for a minimum of 12 hours at 4 °C. After the blocking and staining steps, samples were washed with PBS three times for 20 minutes each and subsequently incubated with commercially available secondary antibodies, diluted in the blocking solution, for 1 hour at room temperature. The slides were then washed with PBS three times for 20 minutes each. A 4% PFA solution was applied as a postfix for 30 minutes. Finally, the slides were washed with PBS three times for 20 minutes each and stored in PBS until imaging.

Immediately before imaging, the storage solution was replaced with a STORM imaging buffer composed of 100 mM Tris pH 9.0, 25 mM NaCl, 20 mM cysteamine (MEA), 70mM β-mercaptoethanol (BME), 3.6mM cyclooctatetraene (COT), 10% w/v glucose, 110 U/mL catalase, and 20 U/mL glucose oxidase. The imaging solution was prepared fresh every week and stored at 4C, with the catalase and glucose oxidase oxygen scavenger system added immediately before imaging. The imaging buffer was covered with a thin layer of mineral oil to reduce diffusion of oxygen into the sample and thereby limit photobleaching. Images were acquired on an Andor iXon Ultra EMCCD camera, with HILO illumination 14. 10000 images of each fluorophore color were collected with 60ms exposure time. The Alexa Fluor 647 channel was imaged first, followed by Alexa Fluor 568-labeled. Single molecule localization and drift correction were performed using the Thunder-STORM plugin of ImageJ 15.

### Establishment of mouse R252A RAW264.7 stable cell lines

Subcloning, lentivirus production, transduction, and cell selection were performed by the University of Michigan Vector Core. A geneblock encoding an amino-terminal HA-tagged mouse Vdac3 cDNA with arginine 252 mutated to alanine, was synthesized by Twist Biosciences. This HA-Vdac3 R252A gene block was then cloned into the NheI/XhoI-digested pLentiLox CAG-mcs-mPGK-meGFP T2a puromycin lentiviral vector. DNA preparation for lentiviral production was carried out using the Qiagen Plasmid Plus Midiprep DNA kit (Qiagen) following the manufacturer's instructions. Lentivirus packaging vectors psPAX2 and the pC1-VSVG were co-transfected with either pLentilox CAG-HA-Vdac3 R252A (for R252A mutant) or mPGK-GFP/puro (for WT) plasmid using standard PEI precipitation methods. PEI precipitation involved incubating the plasmids with PEI (molecular weight 2500, Polysciences, Inc) in OptiMEM (Life technologies) at room temperature for 20 minutes before adding to DMEM with 10% FBS media. This DNA/PEI-containing medium was added to transfect 293T cells (ATCC). Culture supernatant was collected after 72 hours, supplemented with 8 µg/ml polybrene (Sigma), and applied to RAW264.7 cells. The cells were then incubated at 37 °C, 5% CO_2_ for 48 hours before undergoing puromycin selection for 5 days.

### Statistical analysis

The sample size was determined on the basis of experience with similar hypothesis testing experiments. All the data are presented as the means ± SEMs. Statistical analyses were performed using Graph Pad Prism (Version 8.0). Statistical comparisons were made using unpaired two-tailed unpaired t-tests or one-way ANOVA with post hoc correction, as appropriate. Survival curves were analyzed by Kaplan-Meier analysis with the log-rank test. A p-value < 0.05 was considered significant.

## Results

### ScRNA-seq reveals PAD2-dependent macrophage polarization in sepsis

We performed scRNA-seq using the 10× Genomics platform on BALF cells collected from wild-type (WT) and *Pad2^-/-^* mice under sham and PA pneumonia-induced sepsis conditions, yielding a total of 24,480 high-quality cells for analysis (**Fig. 1A**). Uniform Manifold Approximation and Projection (UMAP) dimensionality reduction revealed a dynamic and heterogeneous immune cell landscape across experimental groups **(Fig. 1B)**. AMs were the predominant population in sham-treated mice, while myeloid cells emerged as the dominant population in septic lungs, regardless of genotype. Comparative analysis showed that PAD2 deficiency did not substantially alter the overall immune cell composition in BALF under sepsis (**Fig. 1C**). We performed an unsupervised cluster analysis to investigate the heterogeneity among all myeloid cells. Five transcriptionally distinct subcluster of myeloid cells (Clusters 0-4) were identified.A heterogeneous cell distribution was observed among WT sepsis and *Pad2^-/-^* sepsis groups across these subclusters **(Fig. 1D,E)**. Differential gene expression analysis revealed a distinct transcriptional signature in M2-like anti-inflammatory macrophages from *Pad2^-/-^* septic mice, characterized by significant upregulation of M2 polarization-associated genes, including Arg1, Cd36, Mrc1, Msr1, Il10, and Chil3 **(Fig. 1F)**. These findings suggest that PAD2 plays a regulatory role in modulating macrophage polarization during pulmonary sepsis.

### *Pad2^-/-^* promotes macrophage M2 polarization and metabolic reprogramming in sepsis

To assess the impact of PAD2 on survival outcomes in sepsis, WT and *Pad2^-/-^* mice were monitored for 10 days following intratracheal inoculation with PA. Approximately 50% of the *Pad2^-/-^* mice (n = 8) survived the entire observation period, whereas all WT septic mice (n = 8) succumbed within 3 days post-infection (**Fig. 2A**). Histological evaluation of lung tissues harvested 24 hours post-inoculation, using hematoxylin and eosin (H&E) staining, revealed significantly attenuated acute lung injury (ALI) in *Pad2^-/-^* septic mice compared to WT controls. Blinded histopathological scoring indicated reduced inflammatory cell infiltration, pulmonary edema, and alveolar hemorrhage in the *Pad2^-/-^* group (**Fig. 2B**).

Given our prior scRNA-seq findings of enhanced M2 macrophage signatures in *Pad2^-/-^* BALF cells (**Fig. 1F**), we performed Western blot analysis to quantify protein levels of M2 markers Ym1 and CD206 in BALF cell lysates collected 24 hours post-infection. *Pad2^-/-^* septic mice showed markedly increased expression of Ym1 and CD206 proteins compared to WT septic counterparts (**Fig. 2C**). Consistent with these results, RT-PCR analysis demonstrated elevated mRNA expression of Ym1 and CD206 in BALF cells from* Pad2^-/-^* septic mice (**Supplemental Fig. S1**). Furthermore, ELISA assays revealed significantly higher levels of the anti-inflammatory cytokine TGF-β in BALF from *Pad2^-/-^* septic mice relative to WT septic mice, supporting a shift toward an M2-dominant cytokine milieu (**Fig. 2D**).

Next, BMDMs were isolated from WT and *Pad2^-/-^* mice and stimulated with IL-4 (20 ng/mL) for 48 hours to induce M2 polarization (**Supplemental Fig. S2**). Western blot analysis demonstrated significantly increased expression of CD206 and arginase-1 (Arg1) in IL4 treated *Pad2^-/-^* BMDMs compared to WT controls (**Fig. 2E**).

To evaluate metabolic reprogramming associated with macrophage polarization, we performed Seahorse extracellular flux analysis to measure OXPHOS and glycolysis. Under IL-4 stimulation, *Pad2^-/-^* BMDMs exhibited significantly elevated OCR, indicating enhanced mitochondrial respiration and ATP production via oxidative phosphorylation (**Fig. 2F**). Collectively, these data demonstrate that PAD2 deficiency promotes M2 macrophage polarization and reprograms macrophage metabolism toward an oxidative phenotype during sepsis.

### PAD2 deficiency enhances glutamine metabolism to promote M2 polarization via α-KG

To elucidate the metabolic drivers of M2 macrophage polarization, we performed untargeted metabolomics on BALF cells from WT and *Pad2^-/-^* mice under sham and septic conditions. Heatmap analysis revealed a marked increase in glutamine levels in *Pad2^-/-^* septic BALF cells compared to WT septic mice (**Supplemental Fig. S3**).

Given the established role of glutamine in macrophage polarization 7,16, we quantified glutamine levels in both BALF cells and BMDMs under various conditions. While sepsis generally reduced glutamine in both genotypes, *Pad2^-/-^* septic mice retained significantly higher BALF glutamine concentrations than WT septic mice (**Fig. 3A**), suggesting altered glutamine homeostasis in the absence of PAD2 during sepsis.

To further assess glutamine uptake and metabolism in macrophages, intracellular glutamine levels were measured in BMDMs from WT and *Pad2^-/-^* mice cultured in glutamine-free or glutamine-supplemented media, with or without the glutamine synthase inhibitor L-α-aminoadipic acid (L-α-A). L-α-A treatment reduced intracellular glutamine levels in both genotypes (**Fig. 3B**). To assess the functional consequences of glutamine availability, BMDMs were treated with IL-4 alone, IL-4 plus glutamine, or IL-4 plus glutamine in the presence of L-α-A. Western blot analysis of M2 markers Arg-1 and CD206 demonstrated that intracellular glutamine is required for optimal M2 polarization; L-α-A-mediated glutamine depletion significantly impaired the induction of these markers (**Fig. 3C**).

As M2 polarization is linked to oxidative metabolism, we profiled tricarboxylic acid (TCA) cycle intermediates using targeted metabolomics.* Pad2^-/-^* septic BALF cells showed elevated levels of multiple TCA metabolites compared to WT (**Fig. 3D**), aligning with the increased OCR in IL-4-stimulated *Pad2^-/-^* cells (**Fig. 2F**). Analysis of ScRNA-seq data further demonstrated significantly higher OXPHOS, TCA cycle, and α-KG-related gene module scores in *Pad2*^-/-^ septic BALF cells (**Fig. 3E-G**; gene list in **Supplemental Fig. S4-6**). Consistently, real-time ATP production analysis revealed that PAD2 deficiency shifts BMDMs toward greater mitochondrial ATP dependency (Supplemental Fig. S11), a metabolic state conducive to M2 polarization.

Since glutamine is catabolized into glutamate and subsequently into α-KG, we examined whether glutamine-driven M2 polarization depends on α-KG or its downstream metabolite succinyl-CoA. Using CB-839 (CB, a glutaminase inhibitor) and succinyl phosphonate (SP, a succinyl-CoA synthetase inhibitor) to block distinct steps in the metabolic pathway (**Fig. 3H**), we evaluated Arg-1 expression. Western blot analysis demonstrated that α-KG, but not succinyl-CoA, is critical for promoting M2 polarization (**Fig. 3I**). Dose-response experiments further confirmed that increasing glutamine concentrations led to a corresponding rise in intracellular α-KG levels in BMDMs (**Supplemental Fig. S7**). Together, these results identify α-KG as the key metabolite mediating glutamine-induced M2 macrophage polarization.

To define how PAD2-mediated citrullination influences the metabolic programming of macrophages, we assessed the bioenergetic role of glutamine. we utilized Seahorse extracellular flux analysis to measure OCR and extracellular acidification rate (ECAR) in BMDMs under varying glucose and glutamine conditions. Under glucose-deprived conditions, ECAR was markedly reduced, regardless of glutamine availability, indicating impaired glycolysis (**Fig. 4A**). In glucose-replete media, glutamine supplementation led to a reduced ECAR compared to non-supplemented controls, suggesting a metabolic shift away from glycolysis. Conversely, glutamine supplementation significantly elevated OCR both in the presence and absence of glucose (**Fig. 4B**), highlighting its role in promoting mitochondrial respiration.

Glutamine's effect was unique among amino acids. BMDMs cultured in media containing glutamine (with or without other amino acids) exhibited a significantly higher maximal OCR than those in glutamine-depleted media (**Fig. 4C**). Increasing extracellular glutamine from 0.2 mM to 2 mM significantly elevated the OCR, with a plateau observed at 10 mM (**Fig. 4D**). This dose-dependent enhancement of OCR aligns with levels observed in *Pad2^-/-^* BMDMs, suggesting that *Pad2* deletion enhances glutamine uptake and promotes M2 polarization through glutamine-fueled mitochondrial respiration.

α-KG is a key anaplerotic substrate that fuels the TCA cycle and sustains OXPHOS, processes essential for M2 polarization. Since α-KG is the principal glutamine-derived TCA intermediate 17, we asked if it mediates this bioenergetic effect. Inhibiting glutaminase (and thus α-KG production) with CB-839 impaired mitochondrial function, while inhibiting downstream succinyl-CoA synthesis did not **(Fig. 4E)**. Furthermore, α-KG supplementation alone rescued OCR in a dose-dependent manner in glutamine-free medium **(Fig. 4F)**, confirming its central role.

Collectively, these data establish that glutamine, via its conversion to α-KG, is a critical driver of the mitochondrial OXPHOS required for M2 polarization. Loss of PAD2 activity drives enhanced glutamine flux into α-KG, fueling the TCA cycle and boosting OXPHOS, which collectively create a pro-M2 metabolic environment 7.

### VDAC3 is a novel substrate of PAD2-mediated citrullination in septic macrophages

To investigate whether PAD2-mediated citrullination targets mitochondrial proteins that regulate glutamine metabolism, we conducted in-gel digestion followed by LC-MS/MS analysis of BALF cell lysates from WT and *Pad2^-/-^* mice (**Fig. 5A**). Proteomic and citrullinomic analyses identified VDAC3 as a candidate substrate. Notably, a peptide containing arginine 252 (R252) on VDAC3 was found to be citrullinated in WT septic samples (**Fig. 5B**), but not in *Pad2^-/-^* septic samples (**Fig. 5C**), as evidenced by distinct MS/MS fragmentation patterns (also see **Supplemental Fig. S8**).

Site-specific citrullination of R252 was confirmed by an Ambiguity Score (A-score) of 1000 in WT septic samples (**Fig. 5D**), far exceeding the significance threshold (A-score > 13), establishing that PAD2 enzymatically modifies R252 *in vivo* during sepsis. No citrullination of VDAC3 was observed in BALF cells from either WT sham or *Pad2^-/-^* sham mice.

To validate a physical interaction between PAD2 and VDAC3, we performed co-immunoprecipitation assays in BMDMs stimulated with LPS (200 ng/mL, 24 h). In WT BMDMs, PAD2 and VDAC3 reciprocally co-immunoprecipitated, indicating their interaction under inflammatory conditions. This interaction was absent in *Pad2^-/-^* BMDMs, confirming the specificity and PAD2 dependency of the PAD2-VDAC3 complex (**Fig. 5E**).

These findings establish VDAC3 as a novel PAD2 substrate and identify citrullination at R252 as a sepsis-induced, PAD2-dependent modification. This post-translational modification may impact mitochondrial function by modulating glutamine transport, thereby influencing macrophage metabolic reprogramming and M2 polarization.

### PAD2-mediated citrullination of VDAC3 impairs mitochondrial glutamine transport and M2 macrophage polarization

Given VDAC3's role as a mitochondrial outer membrane channel, we hypothesized that PAD2-mediated citrullination of VDAC3 disrupts mitochondrial glutamine transport. Structural prediction using AlphaFold indicated that residue R252 is located near the C-terminal region, likely within the final β-strand or its adjacent loop (**Fig. 6A**), a region potentially important for channel gating and protein-protein interactions. To verify the increased glutamine entering the mitochondrial following the knockout of *Pad2*, glutamine and mitochondrial were both labeled with antibodies and STORM super-resolution imaging was performed. The result showed that compared to BMDMs from WT mice, BMDMs from *Pad2^-/-^* mice exhibited a significant increase in colocalization of glutamine and mitochondrial (**Fig 6.C**). To assess mitochondrial glutamine uptake, we isolated mitochondria from WT and *Pad2^-/-^* BMDMs and measured radiolabeled [L-[³H] Gln] uptake. Mitochondria from *Pad2^-/-^* BMDMs exhibited significantly increased glutamine uptake at 30 minutes compared to WT controls (**Fig. 6D**), indicating that PAD2 restricts mitochondrial glutamine transport. To directly test the functional role of VDAC3 citrullination, we generated a stable RAW264.7 cell line expressing a VDAC3-R252A mutant via plasmid transfection and lentiviral integration (**Fig. 6B**). This mutation substitutes arginine (R) with alanine (A) at position 252, rendering the site non-citrullinatable. Western blot analysis confirmed expression of HA-tagged VDAC3-R252A in transduced cells (**Fig. 6E**). VDAC3-R252A RAW264.7 macrophages displayed significantly enhanced mitochondrial [³H] glutamine uptake at 30 minutes compared to WT RAW264.7 cells (**Fig. 6F**). Notably, LPS stimulation suppressed glutamine uptake in both WT BMDMs and RAW264.7 cells; however, the *Pad2^-/-^* BMDMs and VDAC3-R252A cells maintained elevated uptake under the same conditions (see **Supplementary Fig. S9**), underscoring a protective effect against inflammatory suppression of glutamine metabolism.

Finally, we stimulated VDAC3-R252A and WT RAW264.7 cells with IL-4 (20 ng/mL, 48 h) to assess the functional consequence of impaired citrullination on M2 polarization. R252A-expressing cells exhibited significantly higher expression of M2 markers CD206 and Arg-1 compared to WT cells (**Fig. 6G**), demonstrating that the non-citrullinatable VDAC3 variant promotes M2 macrophage polarization.

Overall, these data establish that PAD2-mediated citrullination at R252 impairs VDAC3-dependent mitochondrial glutamine transport, thereby restricting OXPHOS and M2 polarization during inflammation.

## Discussion

A hallmark of this complex syndrome is a dysregulated host immune response, characterized by an early hyperinflammatory phase followed by immune paralysis and multi-organ dysfunction 18,19. Macrophages play a central role in this trajectory, with their capacity for polarization into either pro-inflammatory (M1) or anti-inflammatory (M2) phenotypes shaping both pathogen clearance and tissue repair. Metabolic reprogramming underpins this plasticity, with M1 macrophages relying primarily on glycolysis, while M2 macrophages depend on OXPHOS and TCA cycle intermediates such as α-ketoglutarate 3,20,21.

In this study, we uncover a previously unrecognized immunometabolic checkpoint governed by PAD2, which suppresses mitochondrial glutamine uptake and M2 polarization through site-specific citrullination of the outer mitochondrial membrane channel protein VDAC3. We demonstrate that PAD2-mediated citrullination of arginine 252 (R252) on VDAC3 impairs glutamine transport into mitochondria, thereby limiting α-KG generation and blunting the metabolic and epigenetic programs necessary for M2 polarization. Conversely, genetic ablation of PAD2 restores mitochondrial glutamine uptake, enhances OXPHOS, and promotes M2-associated marker expression, conferring survival benefits in murine models of sepsis. The R252A substitution, which prevents citrullination at this critical residue, recapitulates these cellular effects *in vitro*, providing mechanistic evidence that loss of PAD2 enzymatic activity underlies the observed metabolic and phenotypic switch.

Through an integrated strategy employing single-cell RNA sequencing, metabolomics, and mitochondrial flux analysis, we show that PAD2 deficiency reconfigures the macrophage landscape during sepsis. *Pad2^-/-^* mice exhibit upregulation of M2 transcriptional signatures, improved mitochondrial fitness, and enhanced resistance to septic challenge. *In vitro*, PAD2-deficient BMDMs display elevated oxygen consumption and increased expression of CD206 and Arg1, consistent with a metabolic shift toward a reparative, anti-inflammatory state. These findings establish PAD2 as a negative regulator on macrophage polarization and immune resolution during sepsis.

Mechanistically, we identify VDAC3 as a direct substrate of PAD2. Mass spectrometry pinpointed R252, a conserved arginine residue within the C-terminal region of VDAC3, as the site of PAD2-mediated citrullination. This residue lies in proximity to regions implicated in protein-protein interactions and channel regulation. Citrullination of R252, which neutralizes a positively charged guanidino group, likely disrupts electrostatic interactions essential for maintaining VDAC3 conformation or interaction with partner proteins, thereby impeding glutamine flux. This hypothesis is supported by the observation that the VDAC3-R252A mutant, which cannot be citrullinated, restores mitochondrial glutamine uptake and M2 marker expression even in the presence of PAD2.

Our findings reveal that mitochondrial nutrient transport, not just substrate availability or enzymatic control, is a critical node in macrophage immunometabolism. Previous studies have established the role of α-KG in promoting M2 polarization via TCA cycle activity and epigenetic remodeling. We now position PAD2 upstream of this metabolic axis, directly regulating glutamine access to mitochondria through VDAC3. This mechanism represents a paradigm shift in our understanding of how post-translational modifications interface with innate immune metabolism.

Furthermore, our work adds new dimension to the functional repertoire of PAD enzymes in immunity. PAD4, for instance, promotes neutrophil extracellular trap (NET) formation via histone citrullination in the nucleus 22,23. In contrast, PAD2 exerts its function in the cytoplasm and mitochondria, modulating metabolism and macrophage phenotype. This spatial and mechanistic divergence underscores the necessity for isoform-specific PAD inhibitors and reinforces the broader significance of PADs in immune regulation beyond chromatin remodeling.

We acknowledge several limitations. Although VDAC3 emerged as a critical PAD2 substrate in our study, other citrullinated targets may contribute to mitochondrial and immunometabolic dysfunction in sepsis. Moreover, VDAC3 interacts with a host of mitochondrial and cytoplasmic proteins; citrullination at R252 may influence these networks in ways not fully captured in our current assays. In addition, the R252A mutation, while preventing citrullination, may introduce structural changes beyond charge neutralization, future studies employing complementary mutants like R252Q could further dissect the specific contribution of electrostatic versus steric effects. Moreover, Survival improvement was demonstrated in *Pad2^-/-^* mice, not in animals expressing VDAC3-R252A. The R252A mutation provides mechanistic insight at the cellular level and may contribute to the survival phenotype observed in *Pad2*-deficient mice. While our genetic and biochemical data establish the PAD2-VDAC3 axis within macrophages, the global knockout model cannot exclude contributing roles for PAD2 in other cell types. Cell type-specific genetic models (e.g., myeloid-specific *Pad2* knockout mice) would further refine the *in vivo* contribution of PAD2 in macrophages during sepsis. Future studies using lineage-specific approaches will be important to delineate the precise cell type-specific roles of PAD2 in sepsis pathogenesis. Lastly, although our murine models offer robust mechanistic insights, future studies will be essential to confirm the relevance of the PAD2-VDAC3-glutamine axis in human macrophages and sepsis pathology.

Our results also reinforce the central role of glutamine metabolism in macrophage fate decisions. Beyond serving as an anaplerotic substrate, glutamine fuels OXPHOS, supports NADPH generation, and provides precursors for α-KG synthesis-functions indispensable for M2 polarization. Inhibiting glutaminase or blocking α-KG synthesis suppresses M2 gene expression, while exogenous α-KG rescues this defect. These findings support a model in which PAD2 acts as a gatekeeper of glutamine utilization, regulating macrophage phenotype through modulation of mitochondrial substrate flux.

The translational implications of our work are significant. Targeting PAD2 to restore glutamine metabolism and mitochondrial function may represent a promising strategy for reprogramming macrophage responses in sepsis-a disease characterized by an initial surge of inflammation followed by immune collapse. Additionally, biomarkers such as citrullinated VDAC3 or impaired glutamine uptake may help identify patients at risk for immunometabolic dysfunction. Given the broad involvement of macrophages in other diseases, including cancer, fibrosis, and chronic infections, the PAD2-VDAC3-glutamine axis may have therapeutic relevance beyond sepsis.

Our study delineates a novel immune-metabolic circuit in which PAD2 restricts mitochondrial glutamine transport via VDAC3 citrullination, thereby limiting α-KG production and suppressing M2 macrophage polarization. This pathway unveils new therapeutic and diagnostic opportunities for modulating immune metabolism in sepsis and other macrophage-driven pathologies.

## Supplementary Material

Supplementary figures and tables.

## Figures and Tables

**Figure 1:**
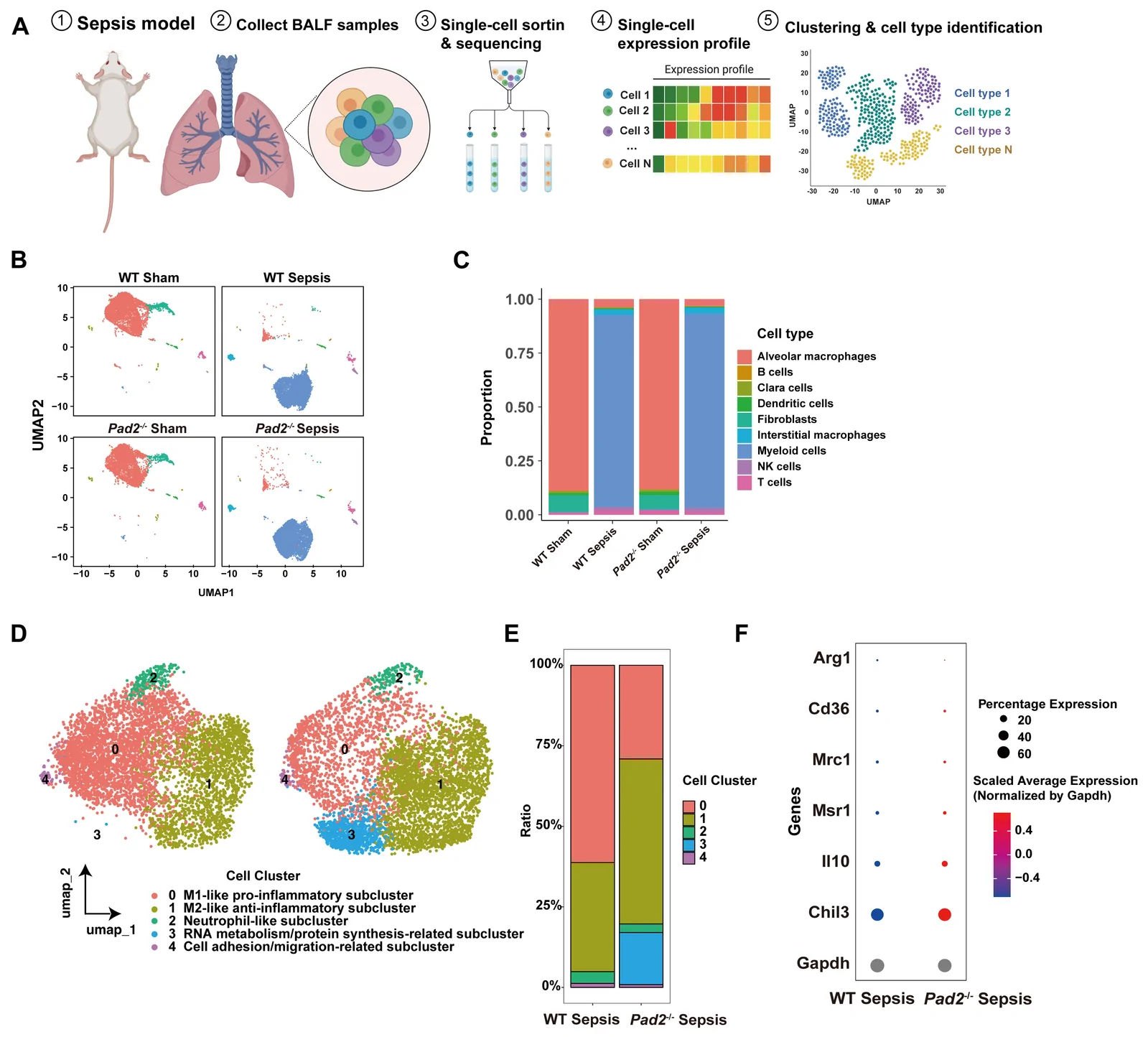
** Single-cell profiling reveals an altered immune landscape in *Pad2^⁻/⁻^* septic mice. A.** Schematic diagram of the experimental workflow for scRNA-seq analysis of BALF cells from WT and *Pad2^-/-^* mice under sham or sepsis conditions. Created in BioRender. **B.** Experimental Group-based UMAP visualization of the WT sham, *Pad2^-/-^* sham, WT sepsis and *Pad2^-/-^* sepsis groups. This visualization highlights the distinct immune landscapes present before and after sepsis, shifting from an alveolar macrophage dominant towards myeloid cell-dominant population. **C.** Distribution of cell-subtype proportions among all the cell populations in each experimental group. **D.** Experimental group-based UMAP visualization of myeloid cells from WT sepsis and *Pad2^-/-^* sepsis groups. **E.** Distribution of cell-subtype proportions among myeloid cell populations in WT sepsis and *Pad2^-/-^* sepsis groups. **F.** Dot plot of normalized expression levels of M2-associated genes (Arg1, Cd36, Mrc1, Msr1, Il10, Chil3) in M2-like anti-inflammatory macrophages from WT and *Pad2*^-/-^ sepsis mice. The expression values were normalized to Gapdh.

**Figure 2:**
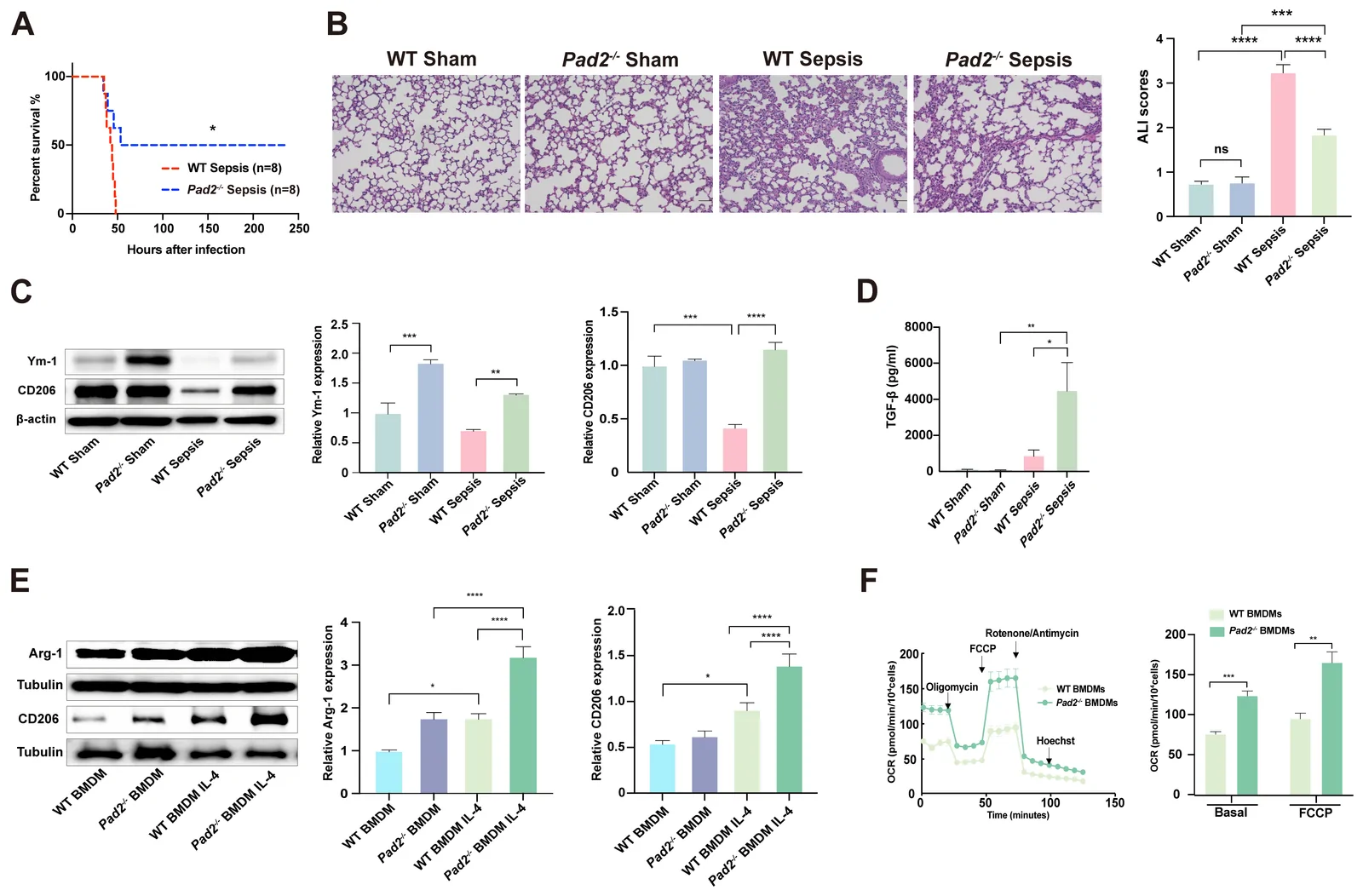
***Pad2* knockout induces M2 macrophage polarization and metabolic reprogramming. A.** Kaplan-Meier survival curves of WT and *Pad2*^-/-^ mice following intranasal inoculation with PA at a dose of 2.5 × 10^6^ CFU per mouse. Survival was monitored for a period of 10 days after inoculation (n = 8 mice/group). **B.** Histopathological analysis of lung injury. The left panel presents H&E-stained lung tissue sections from WT sham, *Pad2*^-/-^ sham, WT sepsis and *Pad2*^-/-^ sepsis mice at 24 hours post-inoculation (n = 4 mice/group). The right panel shows the quantified ALI scores. Scale bars: 50 μm. **C.** Western blot analysis and quantification of Ym1 and CD206 protein levels in BALF cell lysates from WT sham, *Pad2*^-/-^ sham, WT sepsis, and *Pad2*^-/-^ sepsis mice at 24 hours post PA inoculation (n=4). **D.** ELISA quantification of M2-related marker (TGF-β) levels in the BALF of WT sham, *Pad2*^-/-^ sham, WT sepsis, and *Pad2*^-/-^ sepsis mice at 24 hours post PA inoculation (n=5/group). **E.** Western blot analysis and quantification of Arg-1 and CD206 protein expression in WT and *Pad2*^-/-^ BMDMs treated with IL-4 (20 ng/mL, 48 hours) or left untreated (n=6/group). **F.** Oxygen consumption rate (OCR) analysis of WT and *Pad2^⁻/⁻^* BMDMs treated with IL-4 (20 ng/mL, 48 hours). Left: OCRs over time following sequential injections of oligomycin, FCCP, and rotenone/antimycin A. Right: Quantification of basal and FCCP-induced OCRs (n = 4/group). Data were analyzed using unpaired Student's t-tests or one-way ANOVA. The results are presented as means ± standard errors of the means (SEMs). Asterisks (*) denote statistical significance, with P-values indicated as follows: *, P<0.05; **, P < 0.01; ***, P < 0.001; ****, P < 0.0001; ns, not significant.

**Figure 3:**
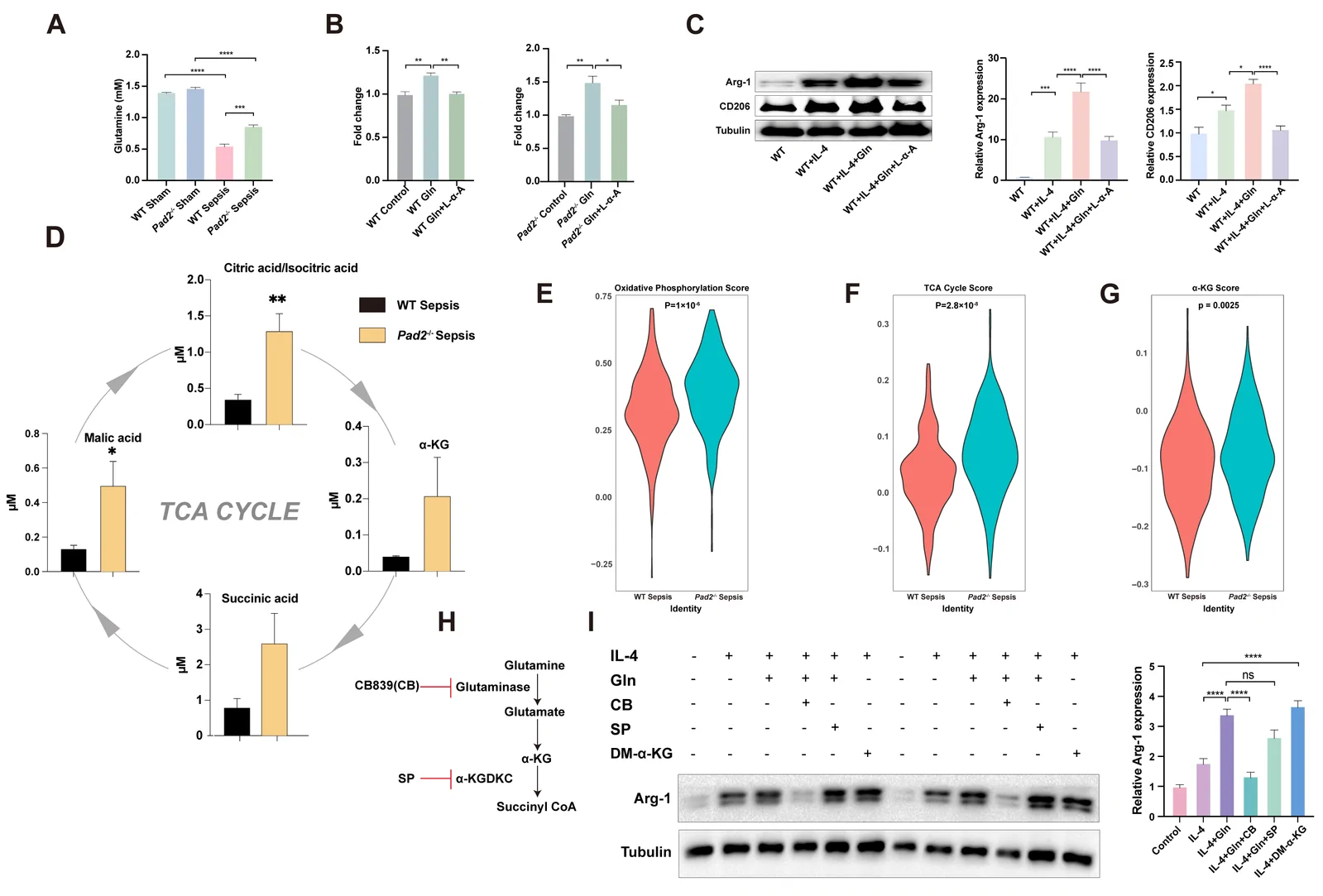
** Glutamine promotes macrophage M2 polarization via its metabolite α-KG. A.** Concentration of glutamine (mM) in BALF cells from WT sham, *Pad2*^-/-^ sham, WT septic, and *Pad2*^-/-^ septic mice (n=4/group). **B.** Intracellular Gln levels in BMDMs from WT and *Pad2*^-/-^ mice. BMDMs were starved of Gln or supplemented with Gln (2 mM), with or without L-α-aminoadipic acid (L-α-A, 200 μM) for 24 h (n=4). Gln, glutamine. L-α-A, an intrinsic glutamine synthase inhibitor. **C.** Western blot analysis of Arg-1 and CD206 expression in BMDMs treated with IL-4 (20 ng/ml) alone, or with Gln (2 mM) for 48 hours or further with L-α-A 200 μM for 24 hours (n=6/group). **D.** MS analysis of TCA cycle metabolites in BALF cells from WT and *Pad2*^-/-^ septic mice. The data are presented as absolute concentrations (n=3-4/group). **E.** OXPHOS-related gene expression module scores in alveolar macrophages (AMs) from septic WT and septic* Pad2*^-/-^ mice, were calculated via AddModuleScore. Scores were visualized with violin plots; significance was determined by the Wilcoxon test. Module scores for OXPHOS-related gene expression were significantly higher in the *Pad2*^-/-^ sepsis group than in the WT sepsis group. The list of OXPHOS-related gene list can be found in **Supplemental Fig S4**. **F.** TCA cycle-related gene expression module scores in alveolar macrophages (AMs) from septic WT and septic* Pad2*^-/-^ mice, were calculated via AddModuleScore. Scores were visualized with violin plots; significance was determined by the Wilcoxon test. Module scores for TCA cycle-related gene expression were significantly higher in the *Pad2*^-/-^ sepsis group than in the WT sepsis group. The list of TCA cycle-related gene list can be found in **Supplemental Fig S5**. **G.** α-KG-related gene expression module scores in alveolar macrophages (AMs) from septic WT and septic* Pad2*^-/-^ mice, were calculated via AddModuleScore. Scores were visualized with violin plots; significance was determined by the Wilcoxon test. Module scores for α-KG-related gene expression were significantly higher in the *Pad2*^-/-^ sepsis group than in the WT sepsis group. The list of α-KG-related gene list can be found in **Supplemental Fig S6**. **H.** Schematic representation of CB-839 (CB) and succinyl phosphonate (SP). **I.** Western blot analysis of Arg-1 expression in BMDMs treated with IL-4 (20 ng/ml), IL-4 + glutamine (2 mM), or IL-4 + DM-α-KG (2 mM), with or without CB (10 μM) or SP (50 μM) for 24 h (n=6). The data were analyzed via the Wilcoxon test or one-way ANOVA. The results are presented as the means ± standard errors of the means (SEMs). Asterisks (*) denote statistical significance, with P-values indicated as follows: *, P<0.05; **, P < 0.01; ***, P < 0.001; ****, P < 0.0001; ns, not significant.

**Figure 4:**
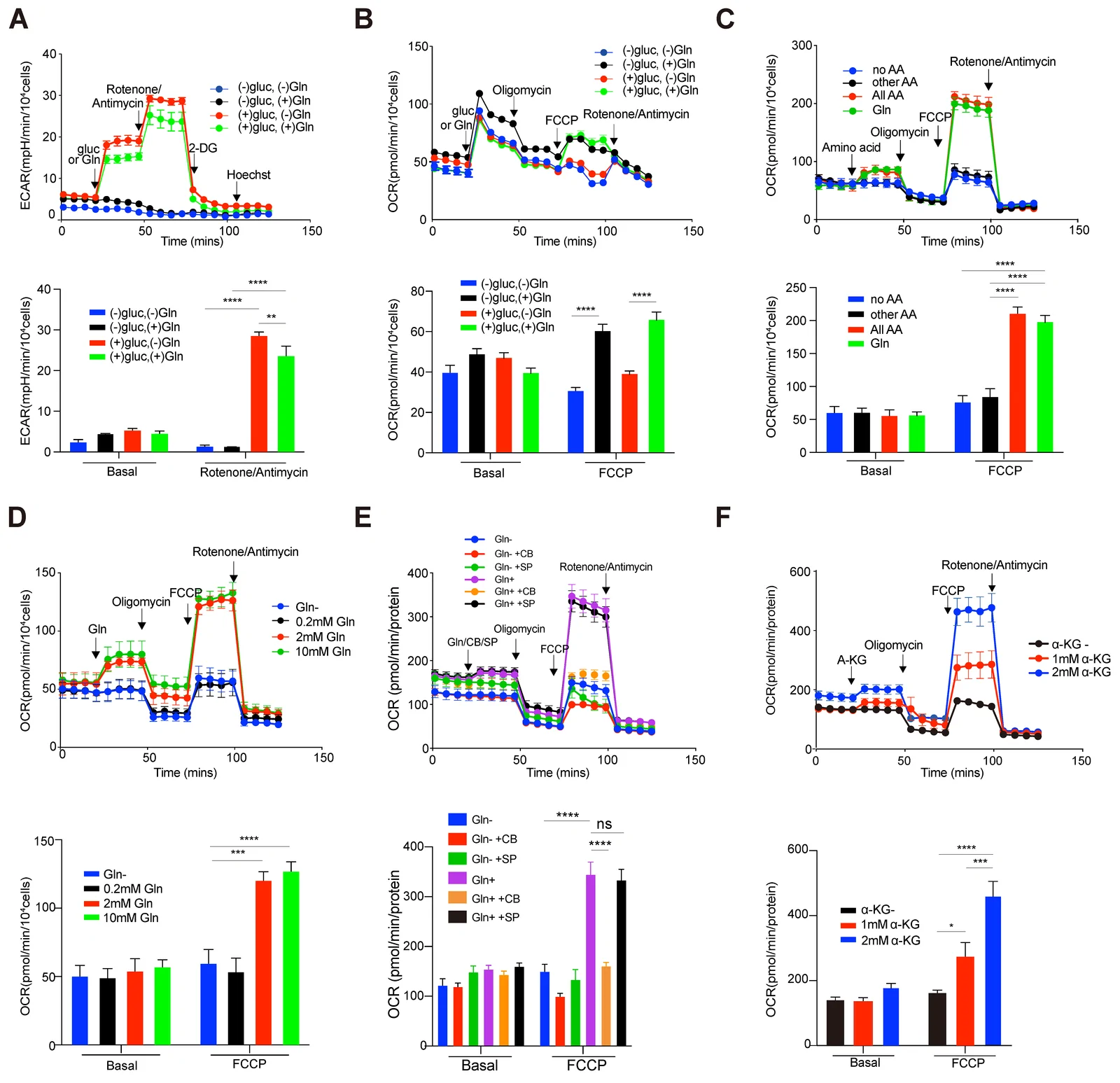
** Glutamine supports mitochondrial respiration and metabolic reprogramming in macrophages. A.** Effects of glucose (gluc) and/or glutamine (Gln) on the extracellular acidification rate (ECAR) in BMDMs plotted over time and bar graphs. **B.** Effects of gluc and/or Gln on the oxygen consumption rate (OCR) in BMDMs plotted over time and bar graphs. **C.** Effects of Gln and other amino acids on the OCR in BMDMs plotted over time and bar graph. **D.** Dose-dependent effects of Gln on the OCR in BMDMs. **E.** Effects of Gln and/or CB and SP on the OCR in BMDMs plotted over time and bar graph. **F.** Effects of different concentrations of α-KG on the OCR in BMDMs plotted over time and bar graph. Bar graph data were analyzed using one-way ANOVA. The results are presented as the mean ± SEM. Statistical significance is indicated as follows: *, P<0.05; ***, P < 0.001; ****, P < 0.0001; ns, not significant.

**Figure 5:**
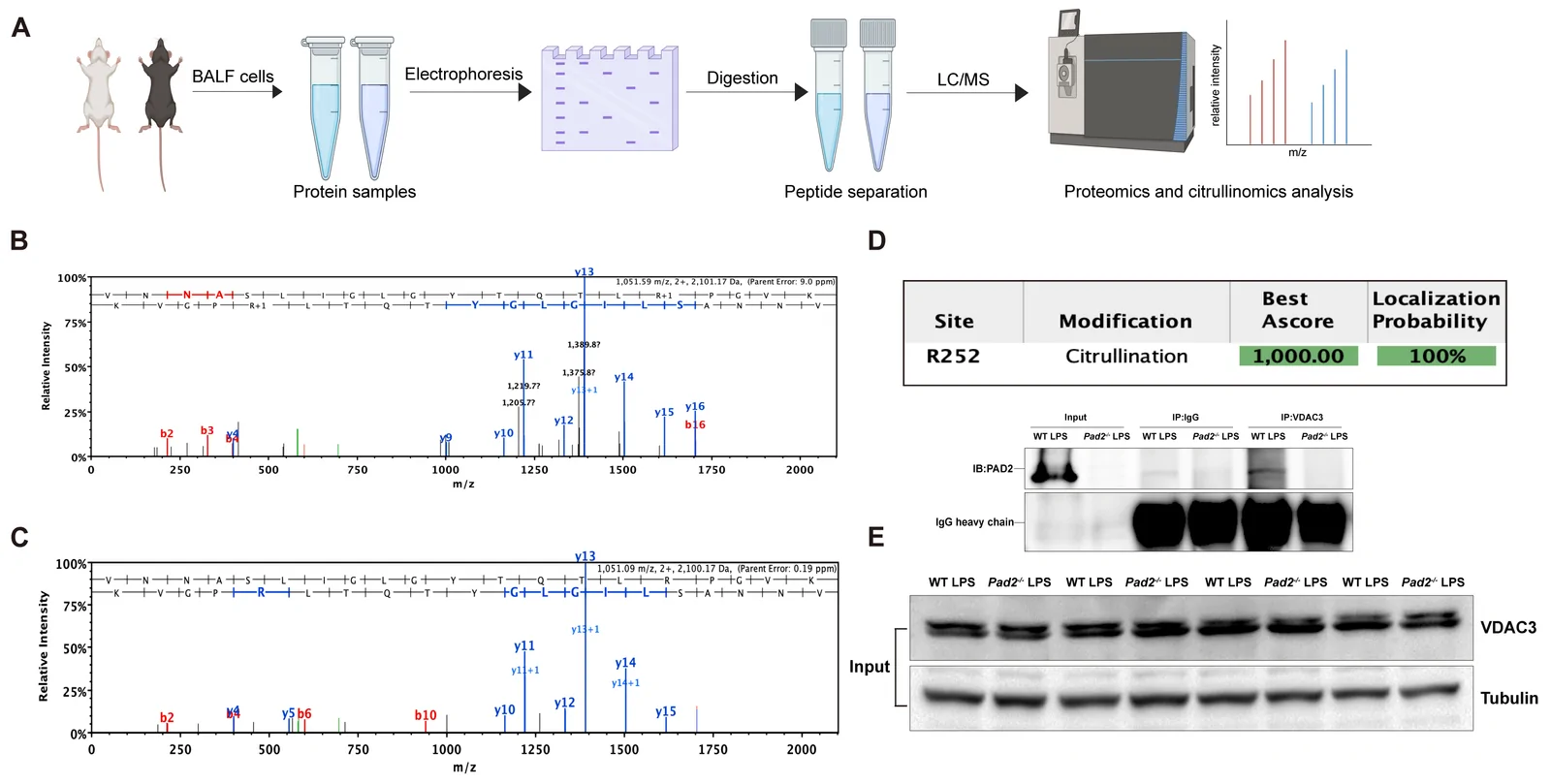
** PAD2 citrullinates VDAC3 at R252 during sepsis. A.** Workflow of citrullinome analysis using BALF cells from WT and *Pad2^⁻/⁻^* mice subjected to *Pseudomonas aeruginosa* (PA)-induced sepsis. Created in BioRender. **B.** The MS spectra of the WT septical group exported from Scaffold PTM software. **C.** The MS spectra of the *Pad2^-/-^* septical group exported from Scaffold PTM software, showing no evidence of R252 citrullination. **D.** Site-specific analysis identified citrullination at R252 in the WT PA group with an A-score of 1000 and 100% localization probability. **E.** BMDMs from WT and *Pad2^-/-^* mice were stimulated with LPS (200 ng/ml, 24 hours), followed by cell lysis and protein extraction. VDAC3 was immunoprecipitated, and PAD2 was detected by Western blot in WT cells, confirming their interaction. Related VDAC3 protein sequences and fragmentation tables also see **Supplemental Fig. S8**.

**Figure 6:**
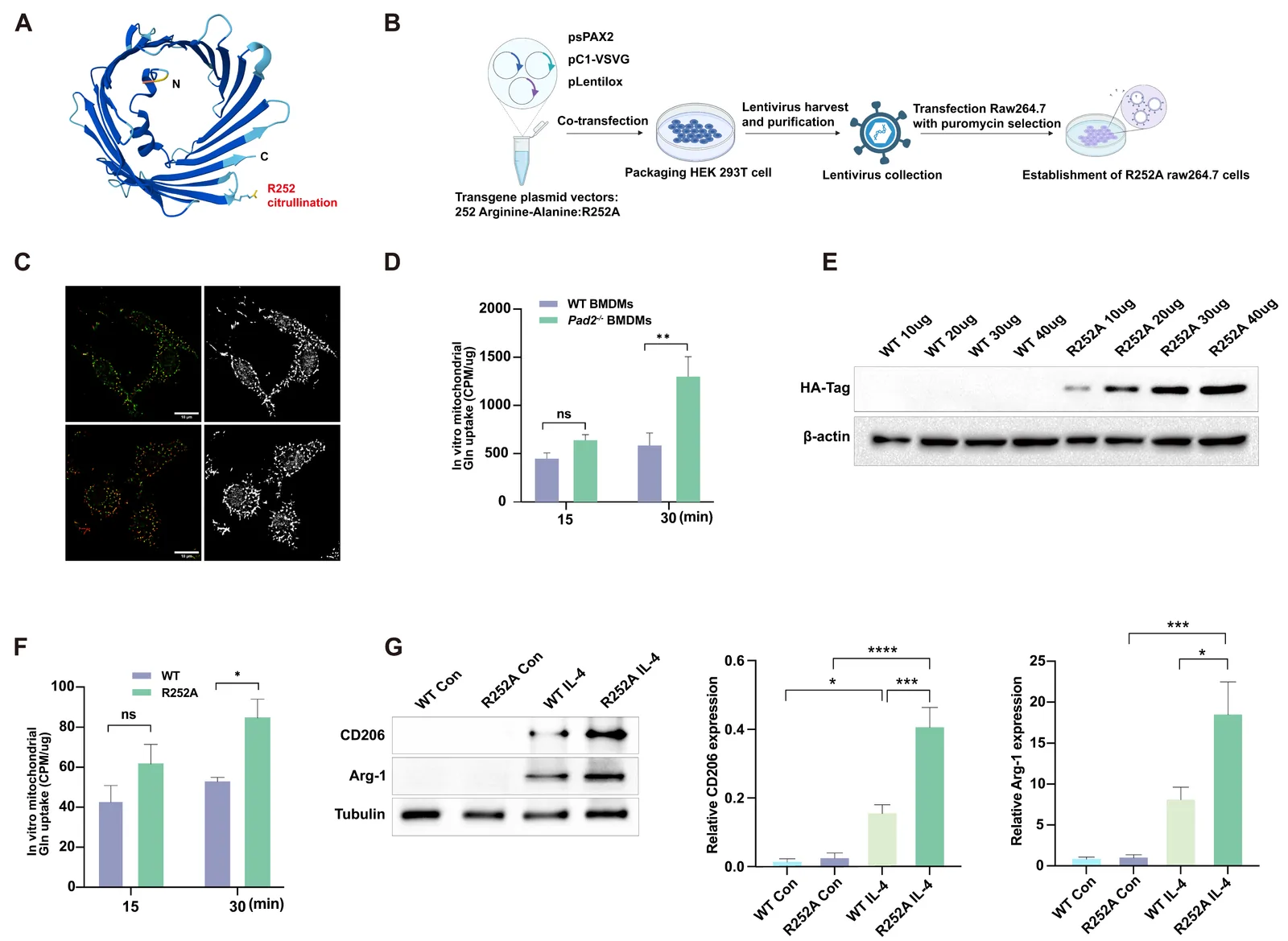
** PAD2-mediated citrullination of VDAC3 impairs mitochondrial glutamine transport and M2 macrophage polarization. A.** The AlphaFold3-predicted 3D structure of VDAC3 with R252 citrullination (N, N-terminal; C, C-terminal). Prediction reliability: blue, very high with pIDDT>90; light blue, confident with 90 > pIDDT > 70; yellow, low with 70 > pIDDT > 50; orange, very low with plDDT < 50. **B.** Schematic showing the generation of RAW264.7 cells expressing the R252A VDAC3 mutant via lentivirus transfection. The process involves using a three-plasmid packaging system to transfect 293T cells, producing and collecting the transgenic lentivirus, and then transducing RAW264.7 cells. Created in BioRender. **C.** Colocalization of glutamine (in red) and mitochondrial (in green) with STORM super-resolution imaging. **D.** [^3^H] glutamine uptake in mitochondria isolated from BMDMs of WT and *Pad2*^-/-^ mice. **E.** Western blot analysis of HA-tagged protein expression in WT and R252A mutant RAW 264.7 macrophages. Lysates from WT and R252A mutant RAW264.7 cells were loaded at increasing amounts (10-40 μg) and analyzed by Western blotting with an anti-HA antibody. β-actin served as a loading control. The results confirmed increased expression of the HA-tagged protein in the R252A mutants and validated the loading consistency. Full uncropped blots are available in supplementary information. **F.** [^3^H] glutamine uptake in mitochondria isolated from RAW264.7 cells transduced with empty vector or R252A mutant. **G.** Western blot analysis of Arg-1 and CD206 expression in RAW264.7 cells transduced with empty vector or R252A mutant, untreated or stimulated with IL-4 for 48 h (n=5). The data from all the bar charts were analyzed via unpaired Student's t tests or one-way ANOVA, as appropriate. The data are presented as the means ± SEMs. Statistical significance is denoted as follows: *, P<0.05; **, P < 0.01; ***, P < 0.001; ns, not significant.

## Data Availability

Data will be made available on request.

## References

[1] Singer M, Deutschman CS, Seymour CW (2016). The third international consensus definitions for sepsis and septic shock (sepsis-3). JAMA.

[2] Hotchkiss RS, Monneret G, Payen D (2013). Immunosuppression in sepsis: a novel understanding of the disorder and. a new therapeutic approach. Lancet Infect Dis.

[3] Murray PJ, Wynn TA (2011). Protective and pathogenic functions of macrophage subsets. Nat Rev Immunol.

[4] O'Neill LA, Kishton RJ, Rathmell J (2016). A guide to immunometabolism for immunologists. Nat Rev Immunol.

[5] Bossche JV, Baardman J, Winther MPJ (2015). Metabolic Characterization of Polarized M1 and M2 Bone Marrow-derived Macrophages Using Real-time Extracellular Flux Analysis. J Vis Exp.

[6] Wang Q, Chen CX, Zuo S (2023). Global research trends on the links between intestinal microbiota and liver diseases: a bibliometric analysis. Am J Transl Res.

[7] Liu PS, Wang HP, Li XY (2017). Alpha-ketoglutarate orchestrates macrophage activation through metabolic and epigenetic reprogramming. Nat Immunol.

[8] Vossenaar ER, Zendman AJ, Venrooij WJ (2003). A growing family of citrullinating enzymes: genes, features and involvement in disease. Bioessays.

[9] Deng QF, Pan BH, Alam HB (2020). Citrullinated Histone H3 as a Therapeutic Target for Endotoxic Shock in Mice. Front Immunol.

[10] Tian YZ, Qu SB, Alam HB (2020). Peptidylarginine deiminase 2 has potential as both a biomarker and therapeutic target of sepsis. JCI Insight.

[11] Dong T, Barasa L, Yu X (2024). AFM41a: A Novel PAD2 Inhibitor for Sepsis Treatment-Efficacy and Mechanism. Int J Biol Sci.

[12] Wu ZY, Li P, Tian YZ (2021). Peptidylarginine Deiminase 2 in Host Immunity: Current Insights and Perspectives. Front Immunol.

[13] Klysz D, Tai XG, Robert PA (2015). Glutamine-dependent alpha-ketoglutarate production regulates the balance between T helper 1 cell and regulatory T cell generation. Sci Signal.

[14] Tokunaga M, Imamoto N, Sakata-Sogawa K (2008). Highly inclined thin illumination enables clear single-molecule imaging in cells. Nat Methods.

[15] Ovesný M, Křížek P, Borkovec J (2014). ThunderSTORM: a comprehensive ImageJ plugin for PALM and STORM data analysis and super-resolution imaging. Bioinformatics.

[16] Jha AK, huang SCC, Sergushichev A (2015). Network integration of parallel metabolic and transcriptional data reveals metabolic modules that regulate macrophage polarization. Immunity.

[17] DeBerardinis RJ, Cheng T (2010). Q's next: the diverse functions of glutamine in metabolism, cell biology and cancer. Oncogene.

[18] Hotchkiss RS, Moldawer LL, Parallels between cancer, infectious disease N Engl J Med. 2014; 371: 380-3.

[19] Poll T, Veerdonk FL, Scicluna BP (2017). The immunopathology of sepsis and potential therapeutic targets. Nat Rev Immunol.

[20] O'Neill LAJ, Artyomov MN (2019). Itaconate: the poster child of metabolic reprogramming in macrophage function. Nat Rev Immunol.

[21] Bossche JV, O'Neill LA, Menon D (2017). Macrophage Immunometabolism: Where Are We (Going)?. Trends Immunol.

[22] Wang YM, Li M, Stadler S (2009). Histone hypercitrullination mediates chromatin decondensation and neutrophil extracellular trap formation. J Cell Biol.

[23] Lewis HD, Liddle J, Coote JE (2015). Inhibition of PAD4 activity is sufficient to disrupt mouse and human NET formation. Nat Chem Biol.

