# Supplementary material for: Citrullination of VDAC3 by PAD2 restricts mitochondrial metabolism and suppresses macrophage reprogramming in sepsis
Source: Int J Biol Sci. 2026 Sep 11;22(15):8302–17. doi: 10.7150/ijbs.126368 (PMC13685606; doi:10.7150/ijbs.126368)
Supplement: Supplementary file 1 [file ijbsv22p8302s1.pdf]

Citrullination of VDAC3 by PAD2 restricts mitochondrial metabolism and suppresses macrophage reprogramming in sepsis

Shao et al.

Address correspondence to: Yongqing Li, Department of Surgery, University of Michigan. NCRC Building 26, Room 363N, 2800 Plymouth road, Ann Arbor, MI 48109. Email address: yqli@med.umich.edu

This file includes:
Supplemental Figure S1-Figure S11
Supplemental Table S1-Table S2

Figure S1.

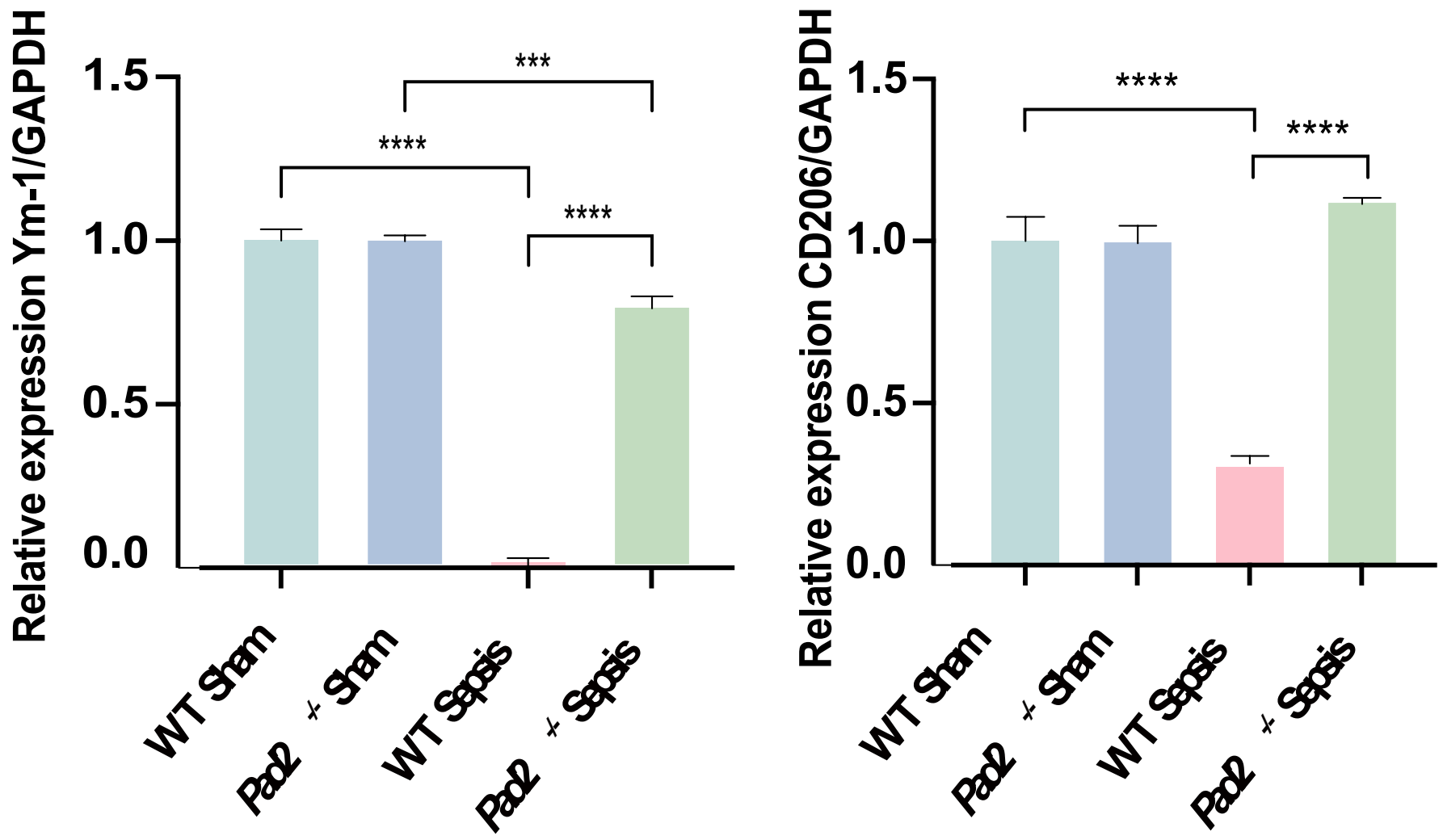

Figure S1. RT-PCR analysis of *Chil3* and *Mrc1* gene expression in BALF cell lysates from WT sham, *Pad2*$^{-/-}$ sham, WT sepsis, and *Pad2*$^{-/-}$ septic mice (n=6/group). Statistical significance is indicated as follows: ***, P < 0.001; ****, P < 0.0001.

Figure S2.

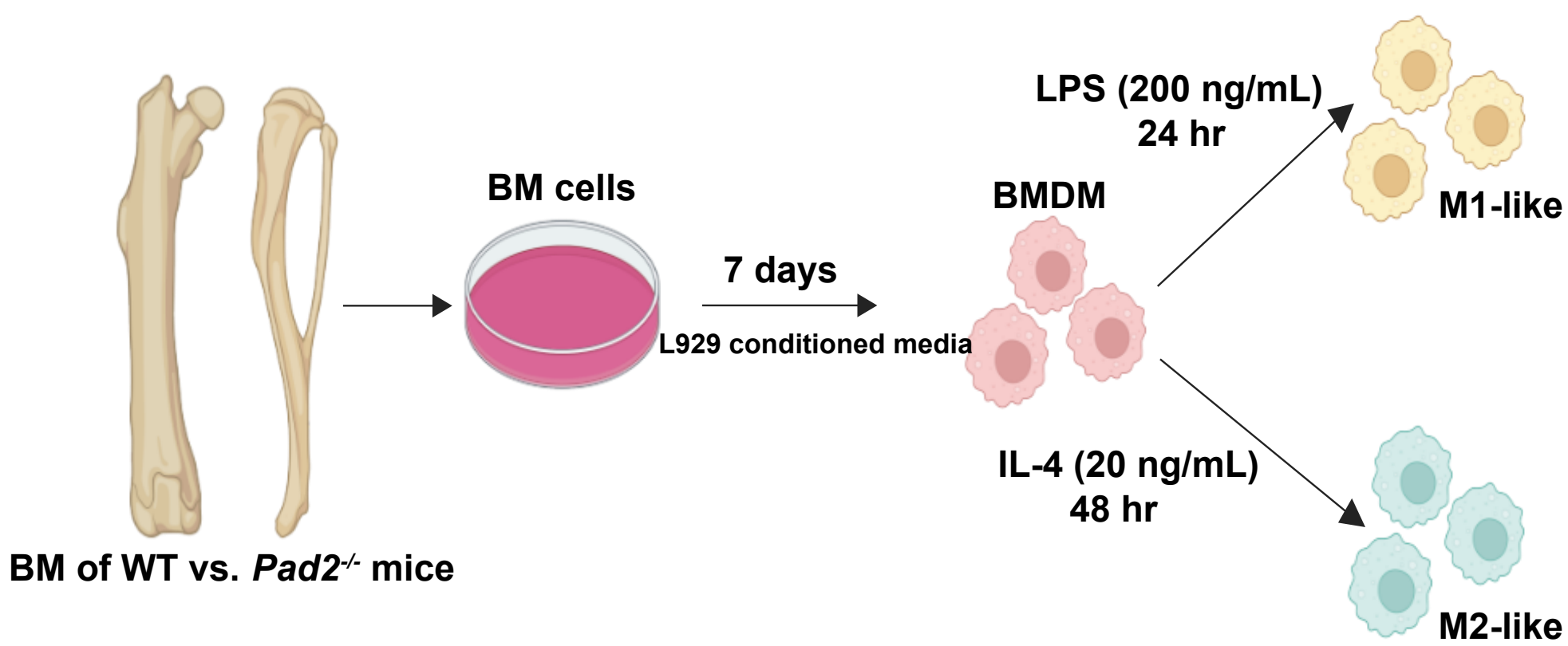

Figure S2. Schematic diagram of mouse BMDM isolation and IL-4–induced polarization protocol. Created in BioRender.

Figure S3.

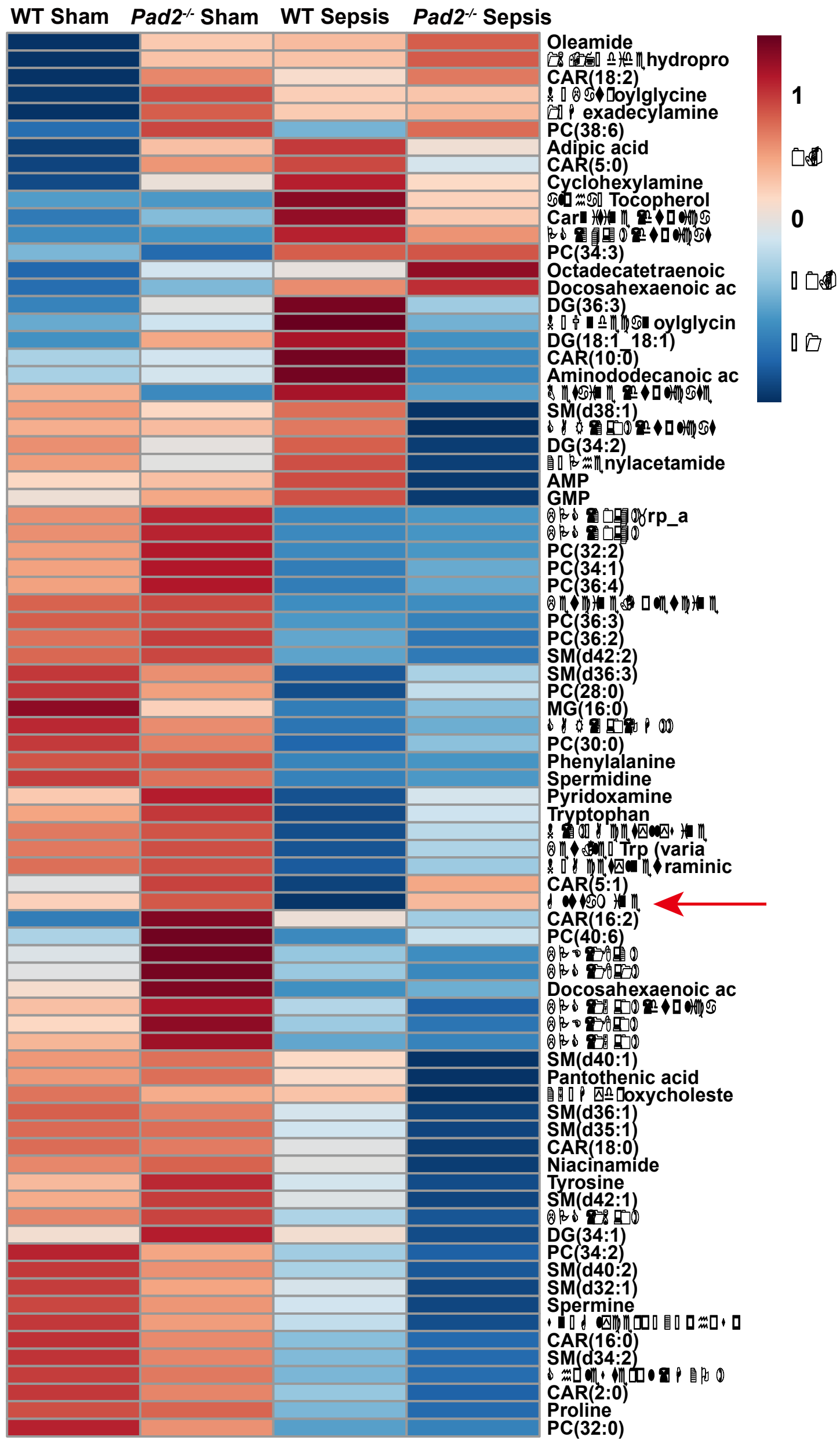

Figure S3. Heatmap showing differentially abundant metabolite expression identified by untargeted metabolomics in BALF cells.

Figure S4.

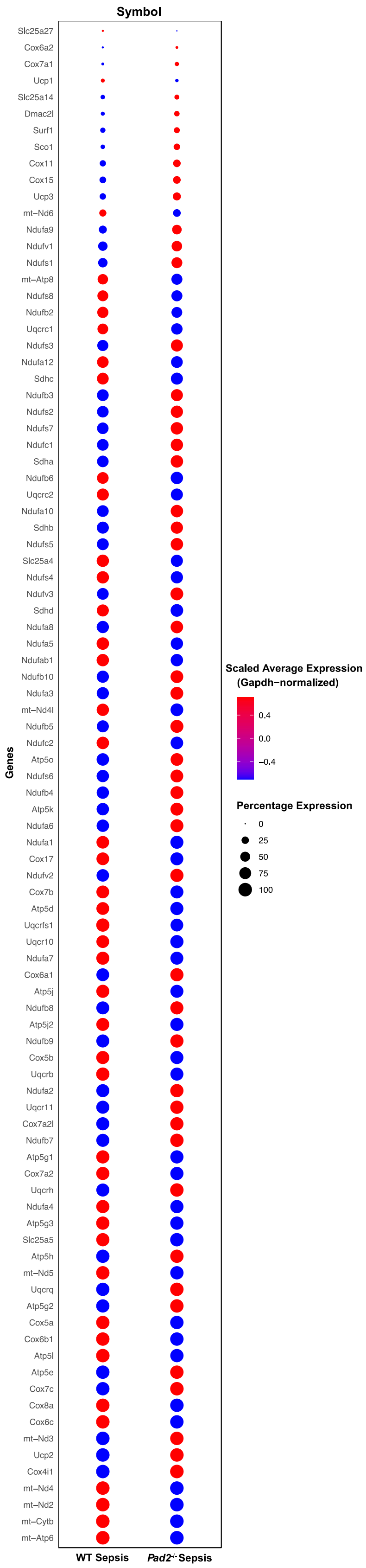
Symbol
Genes
Slc25a27
Cox6a2
Cox7a1
Ucp1
Slc25a14
Dmac2l
Surf1
Sco1
Cox11
Cox15
Ucp3
mt−Nd6
Ndufa9
Ndufv1
Ndufs1
mt−Atp8
Ndufs8
Ndufb2
Uqcrc1
Ndufs3
Ndufa12
Sdhc
Ndufb3
Ndufs2
Ndufs7
Ndufc1
Sdha
Ndufb6
Uqcrc2
Ndufa10
Sdhb
Ndufs5
Slc25a4
Ndufs4
Ndufv3
Sdhd
Ndufa8
Ndufa5
Ndufab1
Ndufb10
Ndufa3
mt−Nd4l
Ndufb5
Ndufc2
Atp5o
Ndufs6
Ndufb4
Atp5k
Ndufa6
Ndufa1
Cox17
Ndufv2
Cox7b
Atp5d
Uqcrfs1
Uqcr10
Ndufa7
Cox6a1
Atp5j
Ndufb8
Atp5j2
Ndufb9
Cox5b
Uqcrb
Ndufa2
Uqcr11
Cox7a2l
Ndufb7
Atp5g1
Cox7a2
Uqcrh
Ndufa4
Atp5g3
Slc25a5
Atp5h
mt−Nd5
Uqcrq
Atp5g2
Cox5a
Cox6b1
Atp5l
Atp5e
Cox7c
Cox8a
Cox6c
mt−Nd3
Ucp2
Cox4i1
mt−Nd4
mt−Nd2
mt−Cytb
mt−Atp6
WT Sepsis
Pad2−/− Sepsis
Scaled Average Expression
(Gapdh−normalized)
0.4
0.0
−0.4
Percentage Expression
0
25
50
75
100

Figure S5.

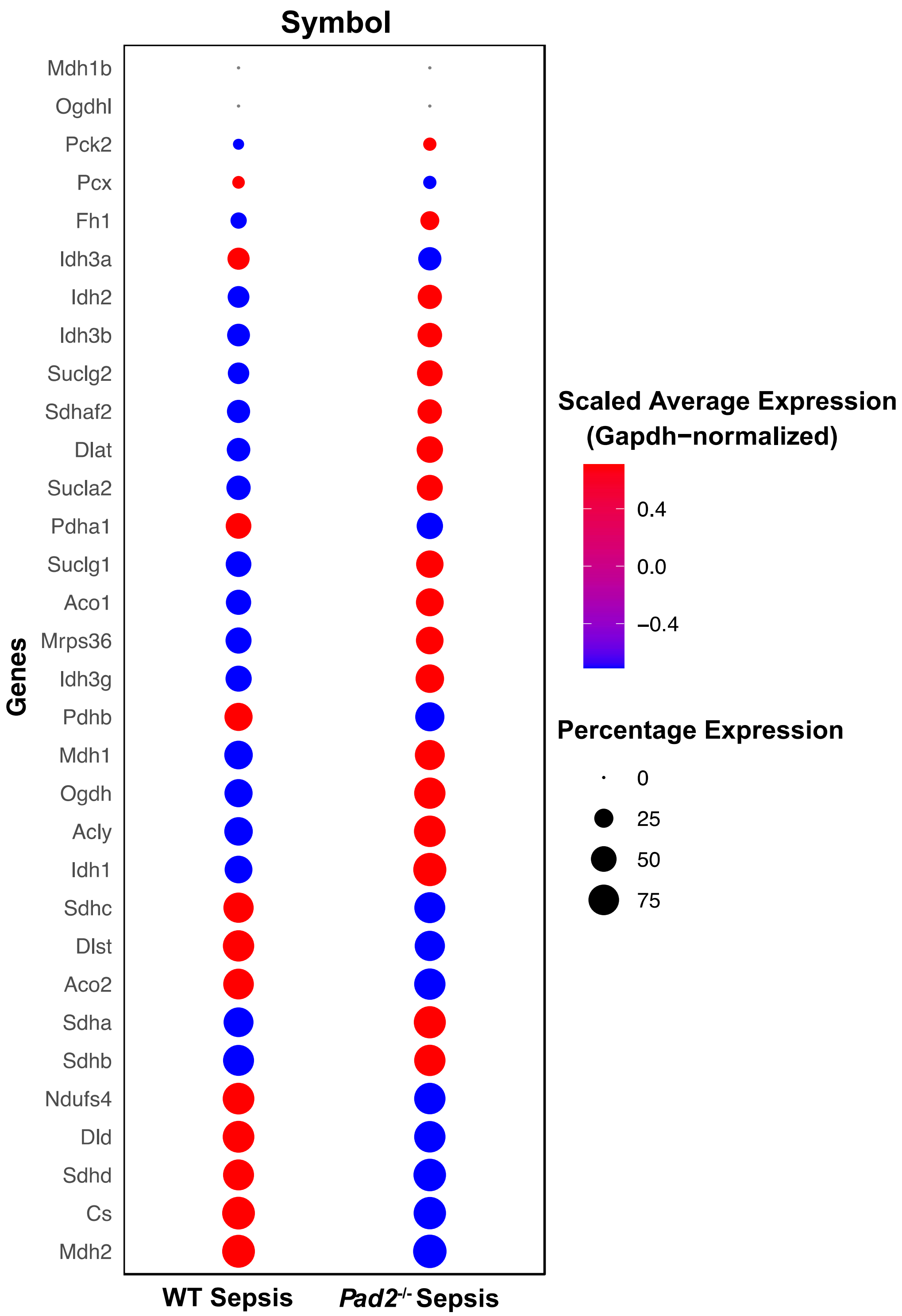
Symbol
Mdh1b
Ogdhl
Pck2
Pcx
Fh1
Idh3a
Idh2
Idh3b
Suclg2
Sdhaf2
Dlat
Sucla2
Pdha1
Suclg1
Aco1
Mrps36
Idh3g
Pdhb
Mdh1
Ogdh
Acly
Idh1
Sdhc
Dlst
Aco2
Sdha
Sdhb
Ndufs4
Dld
Sdhd
Cs
Mdh2
Genes
WT Sepsis
Pad2-/- Sepsis
Scaled Average Expression (Gapdh−normalized)
0.4
0.0
−0.4
Percentage Expression
0
25
50
75

Figure S6.

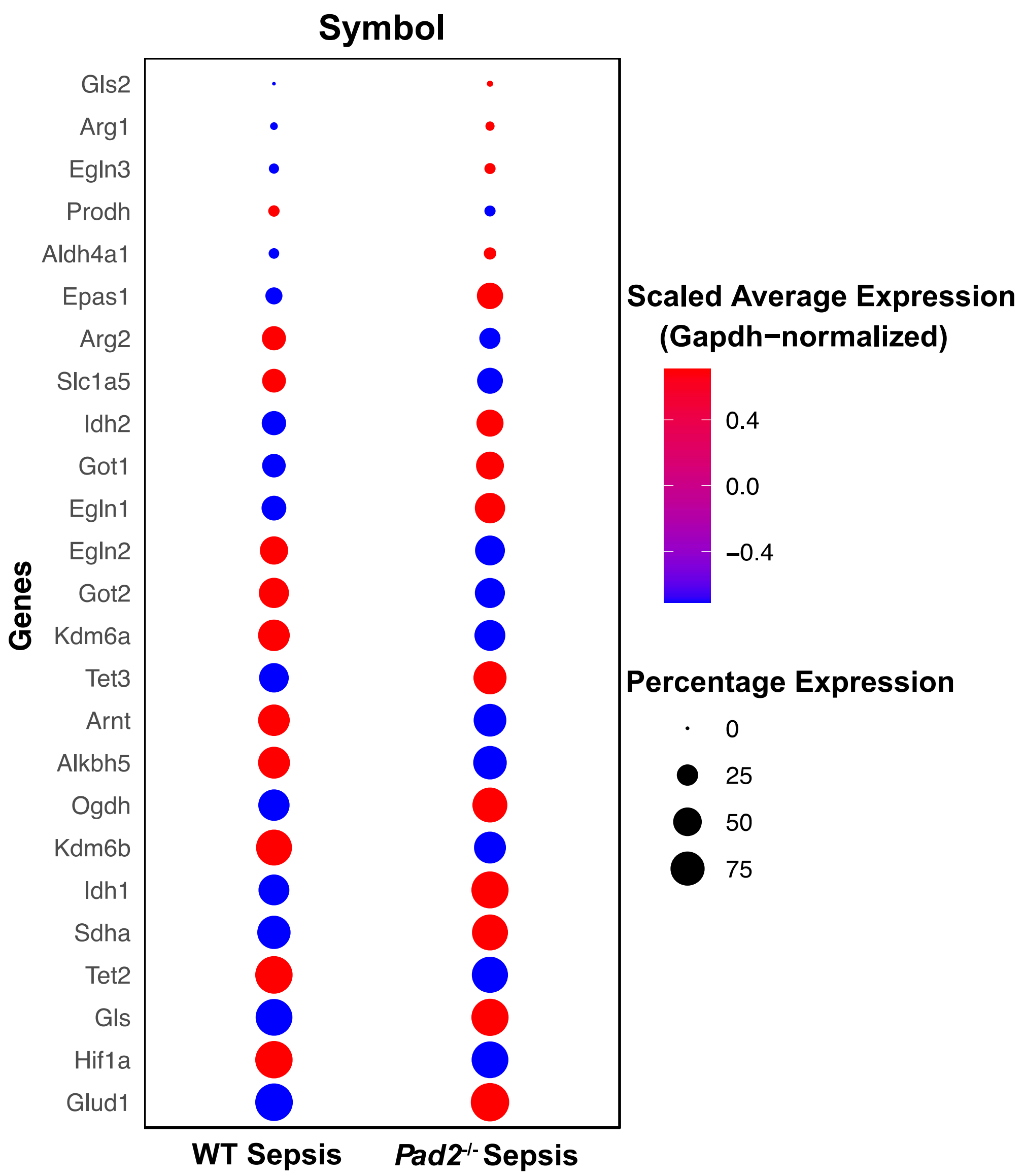

Figure S4-6. Dot plot showing the expression of OXPHOS-related genes (S4), TCA cycle-related genes (S5), αKG-related genes (S6) in alveolar macrophages (AMs) from septic WT and septic *Pad2*$^{-/-}$ mice. Gene expression values were normalized to Gapdh values and scaled across cells. The dot size represents the percentage of cells expressing each gene, while the color indicates the scaled average expression level (red = higher, blue = lower). This analysis highlights differences in OXPHOS

gene expression profiles, TCA cycle-related gene expression profiles and αKG-related gene expression profiles between WT and *Pad2*$^{-/-}$ septic groups.

Figure S7.

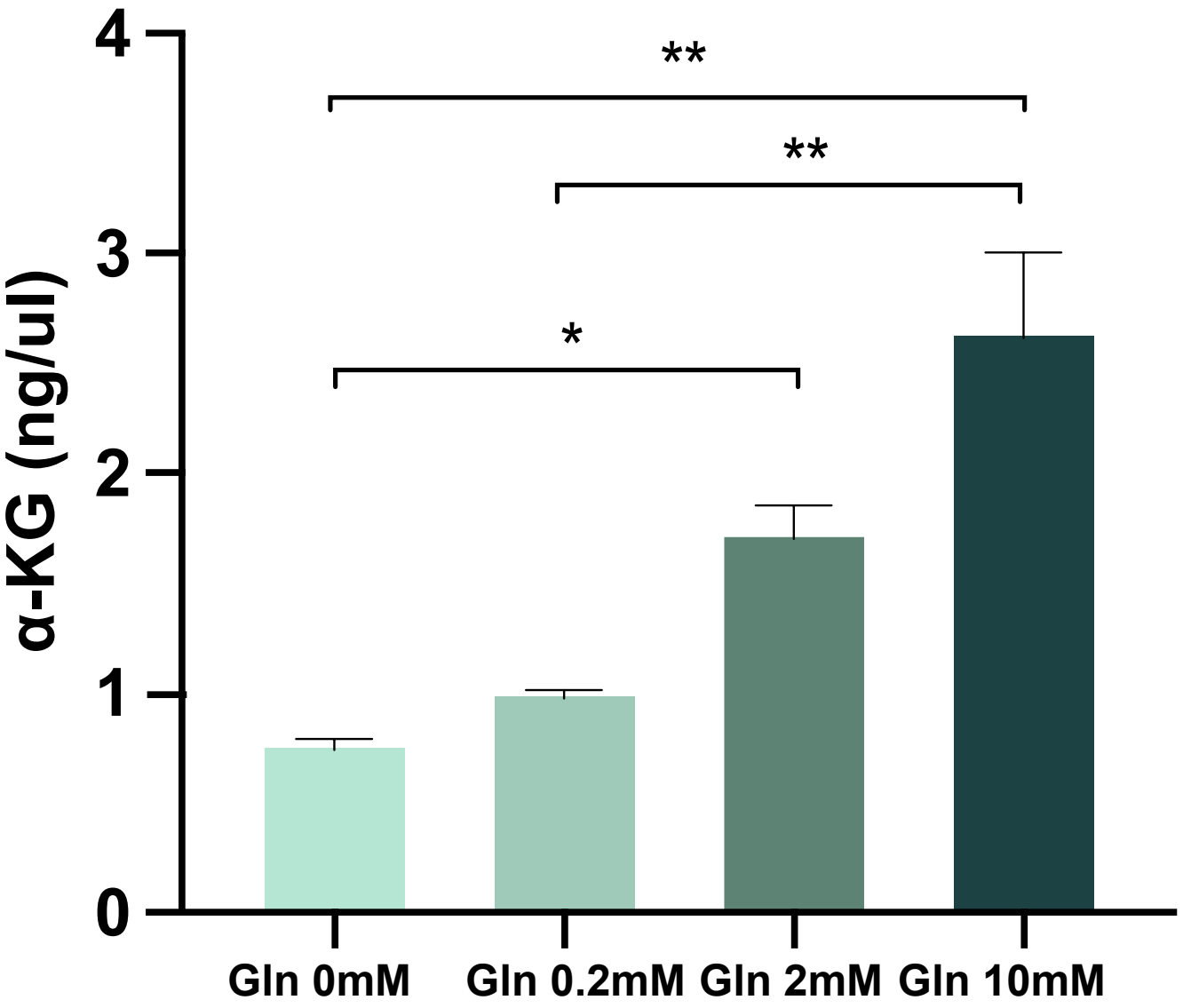

Figure S7. Intracellular αKG levels in BMDMs treated with different concentrations of glutamine (Gln, n=3). Bar graph data were analyzed using one-way ANOVA. The results are presented as the mean ± standard error of the mean (SEMs). Statistical significance is indicated as follows: *, $P<0.05$; **, $P < 0.01$.

Figure S8.

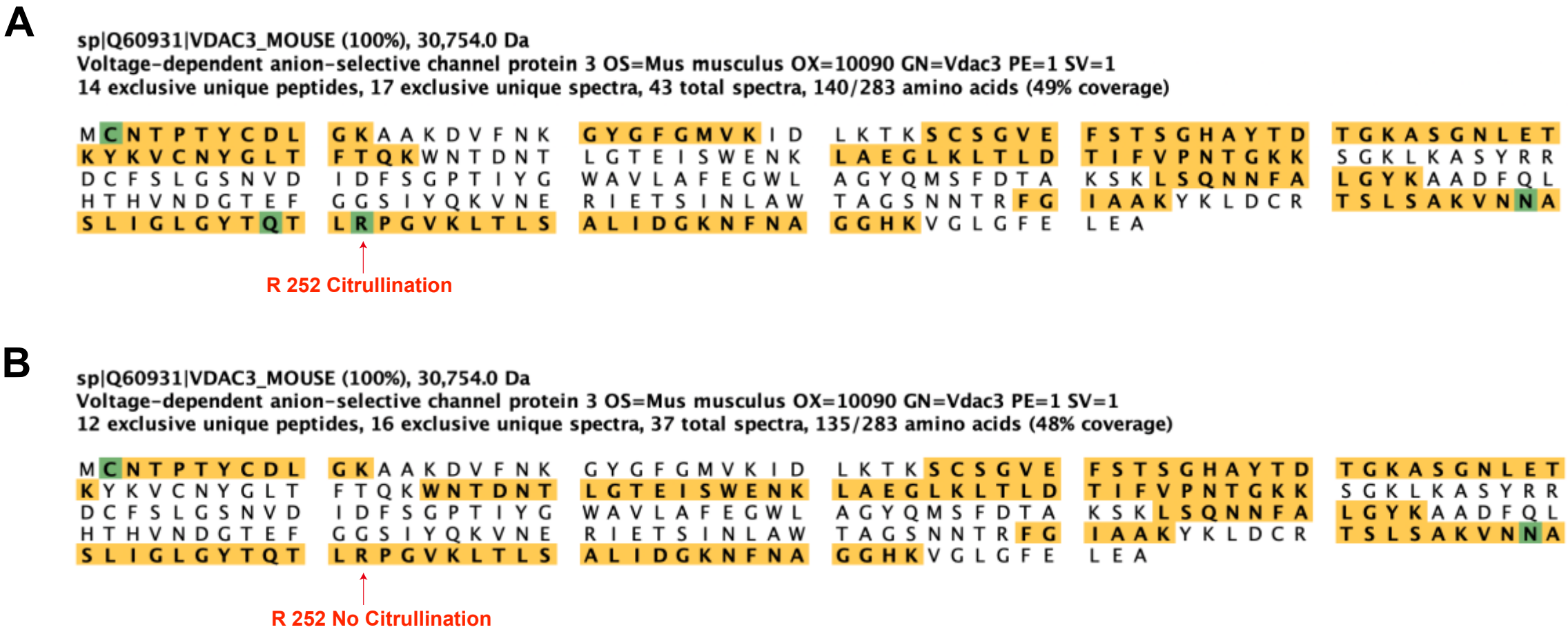

C

B	B Ions	B+2H	B-NH3	B-H2O	AA	Y Ions	Y+2H	Y-NH3	Y-H2O	Y
1	100.1				V	2,102.2	1,051.6	2,085.1	2,084.2	20
2	214.1		197.1		N	2,003.1	1,002.0	1,986.1	1,985.1	19
3	328.2		311.1		N	1,889.0	945.0	1,872.0	1,871.0	18
4	399.2		382.2		A	1,775.0	888.0	1,758.0	1,757.0	17
5	486.2		469.2	468.2	S	1,704.0	852.5	1,686.9	1,686.0	16
6	599.3	300.2	582.3	581.3	L	1,616.9	809.0	1,599.9	1,598.9	15
7	712.4	356.7	695.4	694.4	I	1,503.9	752.4	1,486.8	1,485.8	14
8	769.4	385.2	752.4	751.4	G	1,390.8	695.9	1,373.7	1,372.8	13
9	882.5	441.8	865.5	864.5	L	1,333.7	667.4	1,316.7	1,315.7	12
10	939.5	470.3	922.5	921.5	G	1,220.7	610.8	1,203.6	1,202.7	11
11	1,102.6	551.8	1,085.6	1,084.6	Y	1,163.6	582.3	1,146.6	1,145.6	10
12	1,203.6	602.3	1,186.6	1,185.6	T	1,000.6	500.8	983.6	982.6	9
13	1,331.7	666.4	1,314.7	1,313.7	Q	899.5	450.3	882.5	881.5	8
14	1,432.7	716.9	1,415.7	1,414.7	T	771.5	386.2	754.4	753.5	7
15	1,545.8	773.4	1,528.8	1,527.8	L	670.4	335.7	653.4		6
16	1,702.9	852.0	1,685.9	1,684.9	R+1	557.3	279.2	540.3		5
17	1,800.0	900.5	1,782.9	1,782.0	P	400.3		383.2		4
18	1,857.0	929.0	1,840.0	1,839.0	G	303.2		286.2		3
19	1,956.1	978.5	1,939.0	1,938.0	V	246.2		229.2		2
20	2,102.2	1,051.6	2,085.1	2,084.2	K	147.1		130.1		1

D

B	B Ions	B+2H	B-NH3	B-H2O	AA	Y Ions	Y+2H	Y-NH3	Y-H2O	Y
1	100.1				V	2,101.2	1,051.1	2,084.2	2,083.2	20
2	214.1		197.1		N	2,002.1	1,001.6	1,985.1	1,984.1	19
3	328.2		311.1		N	1,888.1	944.5	1,871.0	1,870.1	18
4	399.2		382.2		A	1,774.0	887.5	1,757.0	1,756.0	17
5	486.2		469.2	468.2	S	1,703.0	852.0	1,686.0	1,685.0	16
6	599.3	300.2	582.3	581.3	L	1,616.0	808.5	1,598.9	1,597.9	15
7	712.4	356.7	695.4	694.4	I	1,502.9	751.9	1,485.8	1,484.9	14
8	769.4	385.2	752.4	751.4	G	1,389.8	695.4	1,372.8	1,371.8	13
9	882.5	441.8	865.5	864.5	L	1,332.8	666.9	1,315.7	1,314.8	12
10	939.5	470.3	922.5	921.5	G	1,219.7	610.3	1,202.7	1,201.7	11
11	1,102.6	551.8	1,085.6	1,084.6	Y	1,162.7	581.8	1,145.6	1,144.6	10
12	1,203.6	602.3	1,186.6	1,185.6	T	999.6	500.3	982.6	981.6	9
13	1,331.7	666.4	1,314.7	1,313.7	Q	898.5	449.8	881.5	880.5	8
14	1,432.7	716.9	1,415.7	1,414.7	T	770.5	385.7	753.5	752.5	7
15	1,545.8	773.4	1,528.8	1,527.8	L	669.4	335.2	652.4		6
16	1,701.9	851.5	1,684.9	1,683.9	R	556.4	278.7	539.3		5
17	1,799.0	900.0	1,782.0	1,781.0	P	400.3		383.2		4
18	1,856.0	928.5	1,839.0	1,838.0	G	303.2		286.2		3
19	1,955.1	978.0	1,938.0	1,937.1	V	246.2		229.2		2
20	2,101.2	1,051.1	2,084.1	2,083.2	K	147.1		130.1		1

Figure S8. PAD2 citrullinates VDAC3 at R252 during sepsis.

A. VDAC3 protein sequence of murine BALF cells from the WT septic group, highlighting citrullination at R252.

B. VDAC3 protein sequence of murine BALF cells from the *Pad2*$^{-/-}$ septic group, showing no citrullination at R252.

C. Corresponding fragmentation table for the WT septic group confirming R252 citrullination.

D. Related fragmentation table of the *Pad2*$^{-/-}$ septic group. Exported from Scaffold PTM software.

Figure. S9

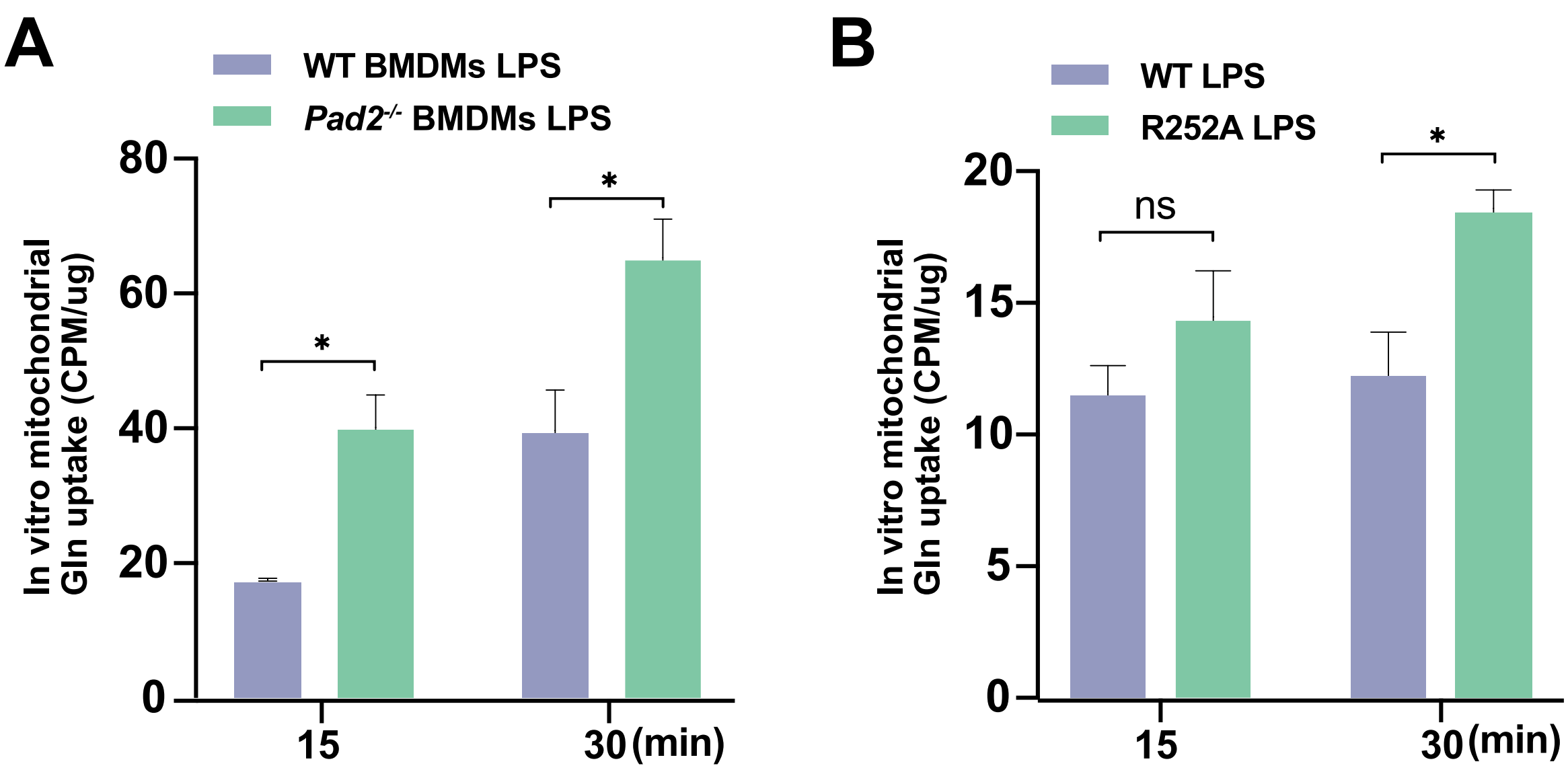

Figure S9. PAD2-mediated citrullination of VDAC3 impairs glutamine uptake in mitochondria isolated from BMDMs under LPS treatment.

A. [^{3}H] glutamine uptake in mitochondria isolated from BMDMs of WT and *Pad2*$^{-/-}$ mice with LPS treatment (200 ng/ml, 24 hours).

B. [^{3}H] glutamine uptake in mitochondria isolated from RAW264.7 cells transduced with empty vector or R252A mutant, with LPS treatment (200 ng/ml, 24 hours).

The data are presented as the means ± SEMs. Statistical significance is denoted as follows: *, P<0.05; ns, not significant.

Figure S10. Transmission Electron Microscopy (TEM) of mitochondria in mouse BALF cells.

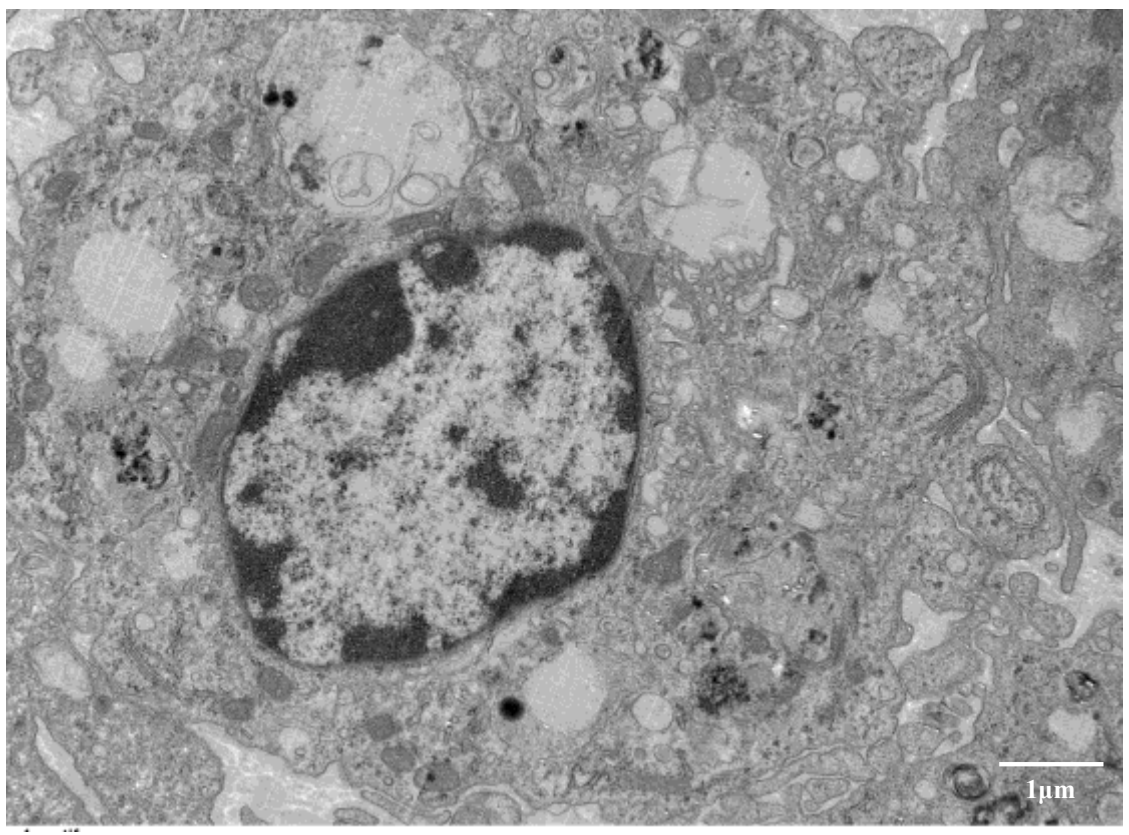

Representative images show that mitochondria in mouse BALF cells maintain normal morphology with intact cristae. Scale bars: 1 μm

Figure S11. PAD2 deficiency reprograms ATP production in bone marrow-derived macrophages (BMDMs) toward enhanced mitochondrial oxidative phosphorylation under M2-polarizing conditions.

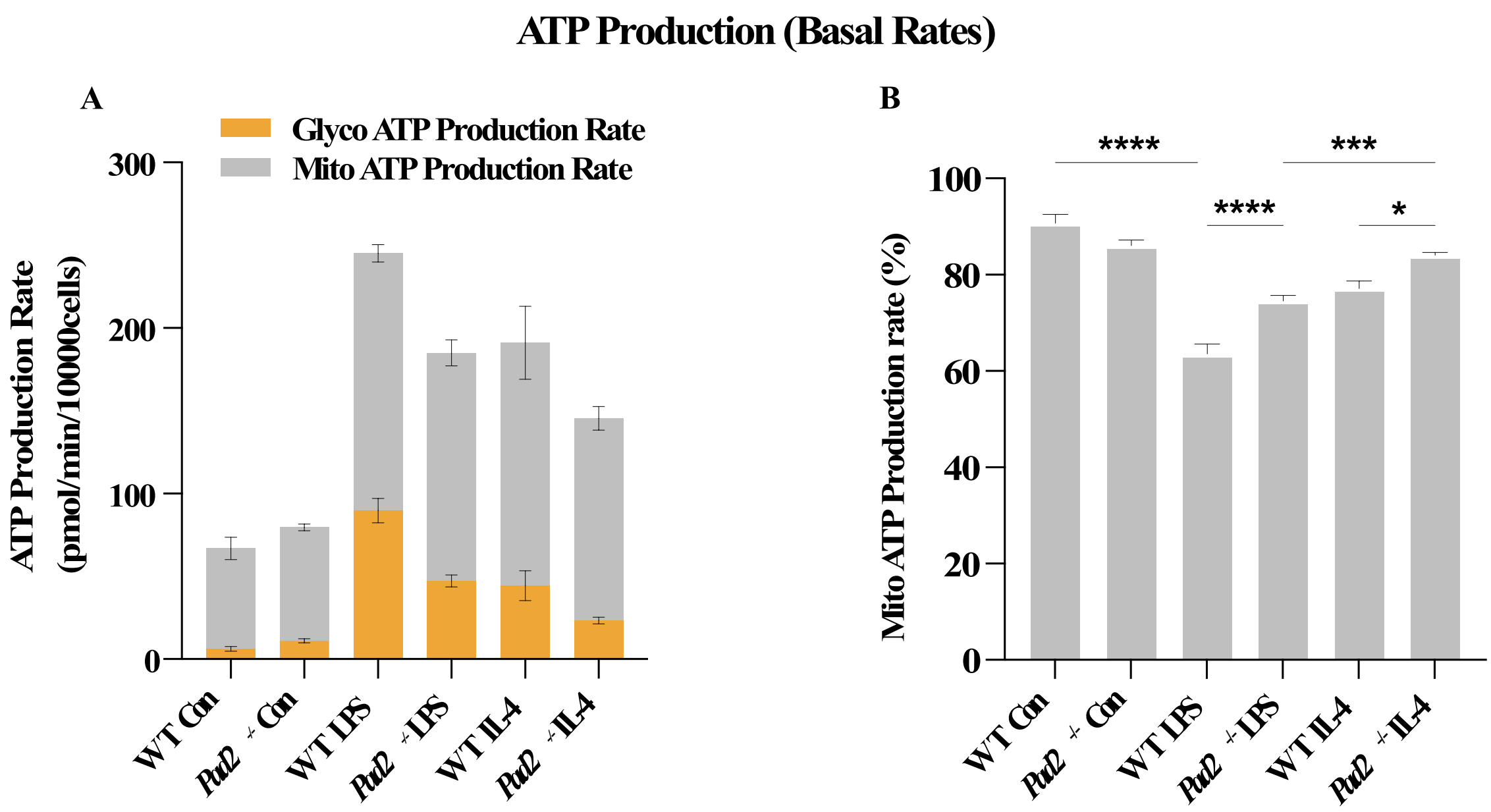

(A) Real-time measurement of ATP production from glycolysis and mitochondria in WT and *Pad2*$^{-/-}$ BMDMs under basal, LPS stimulated (M1), and IL-4 stimulated (M2) conditions.

(B) Quantitative analysis of the proportion of ATP production derived from mitochondria in WT and *Pad2*$^{-/-}$ BMDMs under each condition, calculated from the data in (A).

The data are presented as the means ± SEMs. Statistical significance is denoted as follows: *, $P<0.05$; ***, $P < 0.001$; ****, $P < 0.0001$.

Table S1. Primer sets used in the present manuscript

Gene	Primer	Primer sequence (5′-3′)
GAPDH	F	CACTCACGGCAAATTCAACGGCA
GAPDH	R	GACTCCACGACATACTCAGCAC
IL-1β	F	AGCTTCAGGCAGGCAGTATC
IL-1β	R	AAGGTCCACGGGAAAGACAC
iNOS	F	CAACAGGGAGAAAGCGCAAA
iNOS	R	TGATGGACCCCAAGCAAGAC
Arg-1	F	GGGCTCCTTTCAGGACTAGATA
Arg-1	R	CGAAGCAAGCCAAGGTTAAAG
CD206	F	GGAATCAAGGGCACAGAGTTA
CD206	R	TTCCATCTGCTCCACAATCC

Table S2. Key resources table

REAGENT or RESOURCE	SOURCE	IDENTIFIER
**Antibodies**		
Glutaminase Polyclonal Antibody	Invitrogen	Cat#PA5-85416
Glutamine Synthetase Monoclonal Antibody	Invitrogen	Cat#MA5-27750
Arginase 1 Polyclonal Antibody	Invitrogen	Cat#PA5-29645
Purified Mouse Anti-iNOS/NOS Type II	BD Biosciences	Cat#610328
CD206/MRC1 Rabbit mAb	CST	Cat#24595
GLUL Rabbit mAb	CST	Cat#80636
VDAC3 Polyclonal antibody	Proteintech	Cat#55260-1-AP
Human/Mouse COX4 Antibody	R&D Systems	Cat#MAB6980
Anti-Glutamine antibody	Abcam	Cat#ab9445
α/β-Tubulin antibody	CST	Cat#2148
β-Actin Rabbit mAb	CST	Cat#4970
Normal Rabbit IgG	CST	Cat#2729
PAD2 Polyclonal antibody	Proteintech	Cat#12110-1-AP
PAD2 Monoclonal antibody	Proteintech	Cat#66386-1-Ig
Alexa Fluor® 700 anti-mouse CD45 Antibody	Biolegend	Cat#103128

Mouse CD11c Alexa Fluor® 488-conjugated Antibody	R&D Systems	Cat#FAB69501G
PE-CF594 Rat Anti-Mouse Siglec-F	BD Biosciences	Cat#562757
APC anti-mouse CD38 Antibody	Biolegend	Cat#102711
PE anti-mouse CD206 Antibody	Biolegend	Cat#141705
F4/80 monoclonal Antibody	Invitrogen	Cat#11-4801-82
Human/Mouse Arginase 1/ARG1 PE-conjugated Antibody	R&D Systems	Cat#IC5868P
**Chemicals, peptides, and recombinant proteins**		
Murine IL-4	Sigma	Cat#SRP3211
Murine M-CSF	PeproTech	Cat#315-02
Murine IFN-γ	Sigma	Cat#SRP3058
LPS	Sigma	Cat#L2630
Normal Doney Serum	Jackson ImmunoResearch	Cat#017-000-121
Amino acid free DMEM powder	USBiological	D9800-13
Glucose powder	Sigma	SLBN1246V
MEM Amino acid solution	Gibco	11130051
Sodium bicarbonate powder	Sigma	S6014
DAPI	Sigma	D21490
Hoechst	Thermo Scientific	Cat#62249
^{3}H-Glutamine	Perkin Elmer	Cat#NET-551
UltraComp eBeads™ Compensation Beads	Invitrogen	Cat#01-2222
Seahorse XF calibrant solution	Agilent	Cat#100840-000
Seahorse XF DMEM medium	Agilent	Cat#103575-100
Seahorse XF 1.0 M glucose solution	Agilent	Cat#103577-100
Seahorse XF 100 mM pyruvate solution	Agilent	Cat#103578-100
Seahorse XF 200 mM glutamine solution	Agilent	Cat#103579-100
**Critical commercial assays**		
QuantiTect SYBR Green RT-PCR Kit	Qiagen	Cat#204243
Glutamine Assay Kit	Abcam	Cat#ab197011

Seahorse XF Cell Mito Stress Test kit (OCR)	Agilent	Cat#103015-100
Seahorse XF Real-Time ATP Rate Assay Kit	Agilent	Cat#103592-100
Seahorse XF Glycolysis Stress Test Kit	Agilent	Cat#103020-100
Glutamine Synthetase Activity Assay Kit	Abcam	Cat#ab284572
L-Lactate Assay Kit I	Eton Bioscience	Cat#1200014002
α-Ketoglutarate Assay Kit	Sigma	Cat#MAK054
RNeasy Mini Kit	Qiagen	Cat#74104
Pierce™ Classic Magnetic IP/Co-IP Kit	Thermo Scientific	Cat#88804
Mitochondria Isolation Kit for Cultured Cells	Thermo Scientific	Cat#89874
Pierce™ BCA Protein Assay Kits	Thermo Scientific	Cat#23225
Deproteinizing Sample Preparation Kit	Sigma	Cat#MAK341
Cell Counting Kit-8	DOJINDO	Cat#CK04
Glutamine Elisa Kit	Aviva systems biology	Cat#KI0014
**Critical devices**		
Poly-D-Lysine/Laminin 96-well TC-treated Microplate	Corning	Cat#354596
Pierce™ Protein Concentrators PES	Thermo Scientific	Cat#88513
Seahorse XFe96/XF Pro Cell Culture Microplates	Agilent	Cat#103794-100
**Deposited data**		
Elisa data	Rogel Cancer Center Immunology Core	
Untargeted metabolomics	The Metabolomics Core	
Amino acid analysis	The Metabolomics Core	
Tricarboxylic acid (TCA) analysis	The Metabolomics Core	
Mitochondrial ^{3}H-Glutamine Uptake	University of Michigan Animal Phenotyping Core	
**Experimental models: Cell line**		
RAW264.7	ATCC	N/A
Experimental models: Organisms/strains		
C57BL/6 wild-type mice	The Jackson Laboratory	N/A

Pad2 knockout (*Pad2*$^{-/-}$) mice	Cornell University	N/A
**Software and algorithms**		
FlowJo v.10.9.0	FlowJo	N/A
Prism v.9.1.0	GraphPad	N/A
ImageJ	ImageJ	N/A
Wave v.2.6.3	Agilent	N/A
**Other**		
Seahorse XFe96 Extracellular Flux Analyzer	Agilent	N/A
